# Molecular characterization of pESI-like megaplasmids in *Salmonella* Infantis from poultry in Lebanon

**DOI:** 10.1128/spectrum.02418-25

**Published:** 2026-07-06

**Authors:** Sara Barada, Jose-Rita Gerges, Hadi Hussein, Abdallah Kurdi, Antoine G. Abou Fayad, Rima El Hage, Ghassan M. Matar

**Affiliations:** 1 Department of Experimental Pathology, Immunology and Microbiology, Faculty of Medicine, American University of Beirut 66978, Beirut, Lebanon; 2 Center for Infectious Diseases Research, American University of Beirut 721905, Beirut, Lebanon; 3 World Health Organization (WHO) Collaborating Center for Reference and Research on Bacterial Pathogens 66983 , Beirut, Lebanon; 4 Department of Biochemistry and Molecular Genetics, Faculty of Medicine, American University of Beirut 721910, Beirut, Lebanon; 5 Laboratory of Microbiology, Lebanese Agricultural Research Institute (LARI), Fanar Station, Fanar, Lebanon; University of Pretoria, Pretoria, Gauteng, South Africa; Oita University Hospital, Ho Chi Minh, Vietnam; Center for Veterinary Medicine, US Food & Drug Administration, Rockville, Maryland, USA

**Keywords:** *Salmonella *Infantis, pESI-like megaplasmid, antimicrobial resistance, whole-genome sequencing

## Abstract

*Salmonella enterica* serovar Infantis has emerged as a globally disseminated multidrug-resistant (MDR) pathogen, largely driven by the spread of the plasmid of emerging *Salmonella* Infantis (pESI)-like megaplasmid. In our study, we investigated the prevalence, antimicrobial resistance (AMR) phenotypes, and genomic features of *S*. Infantis isolates collected from poultry farms in Lebanon. A total of 72 isolates were recovered during a nationwide surveillance effort, among which 67 (93%) were MDR based on antimicrobial susceptibility testing (disk diffusion and broth microdilution) results, including resistance to critically important agents such as quinolones, and highly important classes such as tetracyclines and sulfonamides. Whole-genome sequencing was performed on 19 isolates selected through a stratified approach to encompass all identified AMR phenotypes; this analysis revealed a conserved pESI-like backbone together with MDR-associated determinants, including *sul1*, *tet(A*), and *aadA*. Plasmid marker analysis confirmed the presence of pESI in the majority of isolates, with plasmid-associated genes (*ardA* and *trbA*) and replicon markers (IncP and IncFIB(pN55391)) among the most prevalent. Comparative plasmid alignments with representative pESI sequences from Italy, Turkey, and the United States revealed strong conservation of the backbone alongside regional variation in AMR gene content. These findings highlight the role of poultry production systems in Lebanon as reservoirs for pESI-like megaplasmids and MDR *S*. Infantis, underscoring the zoonotic and public health risks posed at the human–animal–environment interface. Strengthened surveillance, antimicrobial stewardship, and biosecurity interventions are urgently needed to mitigate the spread of MDR *S*. Infantis within agriculture and beyond.

**IMPORTANCE:**

The emergence of plasmid of emerging *Salmonella* Infantis (pESI)-like megaplasmids has transformed *Salmonella* Infantis into a globally distributed multidrug-resistant (MDR) clone with the capacity to persist in livestock and disseminate resistance genes across ecological boundaries. Our study provides the first genomic characterization of pESI-positive *S.* Infantis from poultry farms in Lebanon, a region with high antimicrobial usage and limited stewardship frameworks. By integrating phenotypic susceptibility testing and whole-genome sequencing, we demonstrate that Lebanese isolates harbor conserved pESI-like backbone markers together with antimicrobial resistance determinants, aligning them with internationally circulating lineages. Comparative analysis with isolates from Italy, Turkey, and the United States highlights both the evolutionary stability and geographic diversity of pESI. These findings emphasize the urgent need for integrated surveillance and stewardship strategies to curb the spread of MDR *S.* Infantis and reduce the zoonotic risk at the human–animal–environment interface.

## INTRODUCTION

The global emergence and spread of multidrug-resistant (MDR) *Salmonella enterica* serovars represent a significant public health and food threat. Among these, *Salmonella* Infantis has emerged as a particularly concerning serovar due to its increasing prevalence in poultry and its association with MDR phenotypes (1, 2). In Lebanon, surveillance of the poultry production chain reported that *S.* Infantis accounted for 32.9% of the isolates, making it one of the most prevalent serovars in this sector (3).

Lebanon occupies a strategic geographic position at the intersection of Asia, Africa, and Europe and acts as a regional hub for trade and population mobility, making it an important sentinel setting for monitoring the emergence and spread of antimicrobial resistance (AMR) across continents.

The rise of MDR *S*. Infantis is often linked to the acquisition of large plasmids, such as the plasmid of emerging *Salmonella* Infantis (pESI)/pESI-like megaplasmids, which carry a range of antimicrobial resistance determinants and have been increasingly reported among poultry-associated *S*. Infantis lineages (1, 4, 5). These include genes conferring resistance to critically important antibiotics such as quinolones, as well as highly important agents like tetracyclines and sulfonamides, as per the World Health Organization’s (WHO) classification (6). Beyond antimicrobial resistance, pESI-like plasmids frequently encode tolerance to heavy metals and antiseptics, further enhancing bacterial survival under agricultural pressures (1, 7). Collectively, this genomic arsenal reinforces the ecological success of *S*. Infantis and supports its zoonotic transmission potential (1, 8).

Globally, pESI-positive *S*. Infantis has been increasingly reported in poultry production systems, with dissemination documented across Europe, the Mediterranean basin, the Middle East, and North America (1, 9–11). Similar patterns have been reported in North America, where McMillan et al. further demonstrated that the high prevalence of poultry-associated *S*. Infantis was associated with clonal expansion of a lineage carrying the pESI-like plasmid (5, 12). Reports from the Eastern Mediterranean have also described pESI-positive *S*. Infantis lineages, further supporting the regional relevance of this megaplasmid and the need to contextualize Lebanese isolates within broader regional and global pESI surveillance frameworks (13). This high propensity for global dissemination is attributed to the carriage of the pESI-like megaplasmid, which contributes to the adaptability and persistence of *S*. Infantis in agricultural environments (14). In Lebanon, the emergence of MDR pathogens, including *Salmonella*, has been reported in both agricultural and clinical settings, reflecting the extensive usage of antibiotics in livestock and weak antimicrobial stewardship (15, 16). For instance, a recent survey reported resistance rates exceeding 90% for tetracyclines and sulfonamides among poultry-derived MDR *Salmonella* isolates (17). Such findings highlight the urgent need for genomic surveillance to trace the drivers of resistance and their mobility across food-animal reservoirs.

In our study, we examined the prevalence, AMR profiles, and genomic features of *S*. Infantis isolates from poultry farms in Lebanon. By combining phenotypic susceptibility testing and whole-genome sequencing (WGS), we explored the role of the pESI-like megaplasmid in driving resistance and persistence. Our findings demonstrate high resistance rates to critically important antibiotics, such as quinolones, as well as highly important agents like tetracyclines and sulfonamides, according to the WHO classification (6).

Additionally, we contextualized the genomic architecture of Lebanese pESI-like plasmids through comparative analysis with reference plasmids from Italy, Turkey, and the United States. This approach provides insights into the evolutionary conservation and international dissemination of these megaplasmids. To our knowledge, this represents the first genomic characterization of pESI-positive *S*. Infantis in poultry in Lebanon, providing insights into AMR dissemination at the human–animal–environment interface.

## MATERIALS AND METHODS

### *Salmonella* Infanti*s* isolates

A total of 72 *Salmonella enterica* serovar Infantis isolates were collected in March 2017 from multiple poultry farms located in the Mount Lebanon region, as part of a nationwide agricultural surveillance campaign targeting zoonotic pathogens in livestock. All isolates were collected during a single sampling period (March 2017), providing a cross-sectional snapshot of *Salmonella* Infantis circulating in Lebanese poultry farms at that time. All samples were obtained from fecal matter and cloacal swabs collected directly from live poultry during farm-level visits. However, detailed farm-level metadata, including the exact number of farms sampled, individual farm identifiers, and production types, such as broiler or broiler-breeder farms, were not available for the present analysis. Due to the absence of farm-level metadata, isolates were analyzed collectively at the regional level (Mount Lebanon). This governorate represents one of Lebanon’s primary poultry production hubs, providing a representative baseline for AMR in the sector. As a result, downstream phylogenetic clustering could not be attributed to specific farms or production types, and relatedness among isolates was interpreted as reflecting circulation within the broader regional poultry production network, with possible contribution from shared upstream sources such as hatcheries or feed suppliers. Following collection, the isolates were transported to the Lebanese Agricultural Research Institute (LARI) for initial processing, then transferred to the Bacteriology and Molecular Microbiology Laboratory at the American University of Beirut for further phenotypic and genomic analysis.

### Antimicrobial susceptibility testing

Antimicrobial susceptibility testing was performed using disk diffusion and broth microdilution, following the Clinical & Laboratory Standards Institute (CLSI) M100 ED34:2024 guidelines (18). However, since CLSI did not provide breakpoints for cefoxitin, gentamicin, amoxicillin-clavulanic acid, and nalidixic acid, we referred to the European Committee on Antimicrobial Susceptibility Testing (EUCAST) for these antibiotics (19). Quality control was ensured using *Escherichia coli* ATCC 25922 as the reference strain for both testing methods, in accordance with CLSI and EUCAST guidelines.

For the disk diffusion method, eight antimicrobials were tested: ceftriaxone (CTR, 30 µg), cefoxitin (CXI, 30 µg), meropenem (MER, 10 µg), ampicillin (AMP, 10 µg), chloramphenicol (CHL, 30 µg), gentamicin (GEN, 10 µg), ciprofloxacin (CIP, 5 µg), and tetracycline (TET, 30 µg). Plates were then incubated at 37°C for 18–24 h, after which the diameters of the inhibition zones were measured and interpreted using standard CLSI and EUCAST breakpoints.

Further antimicrobial testing was conducted using the broth microdilution method against four additional antibiotics: trimethoprim-sulfamethoxazole (TRS), amoxicillin-clavulanic acid (AMC), azithromycin (AZI), and nalidixic acid (NAL). Twofold serial dilutions were performed with concentrations ranging from 2,048 µg/mL to 0.5 µg/mL and incubated at 37°C for 18–24 h to determine minimum inhibitory concentrations (MICs) in duplicate. Specific resistance breakpoints for broth microdilution assays were defined as follows: nalidixic acid (MIC ≥ 32 µg/mL), trimethoprim-sulfamethoxazole (MIC ≥ 4/76 µg/mL), amoxicillin-clavulanic acid (MIC ≥ 32 µg/mL), and azithromycin (MIC ≥ 32 µg/mL). Isolates that exhibited resistance against three or more antimicrobial classes through either method were classified as MDR.

### Whole-genome sequencing

WGS was performed on 19 MDR *S*. Infantis isolates selected from the collection of 72 to capture the diversity of antimicrobial susceptibility phenotypes observed. Selection criteria included representation of different AST patterns, including the predominant MDR phenotype (e.g., TET-TRS-NAL) as well as less frequent resistance combinations involving quinolones, β-lactams, aminoglycosides, phenicols, and amoxicillin-clavulanic acid (Table S1). Sequencing was carried out at the Thakur Molecular Epidemiology Laboratory, North Carolina State University, College of Veterinary Medicine. Prior to sequencing, fresh cultures of each isolate were grown on MacConkey agar, and genomic DNA was extracted using QIAamp DNA Mini Kit (Qiagen, Valencia, CA, USA). Sequencing libraries were prepared using the Nextera XT Library Prep Kit (Illumina GmbH, Munich, Germany), and sequencing was performed using the Illumina MiSeq platform with paired-end reads (2 × 150 bp).

### Bioinformatic analysis

Reads quality control and trimming were performed using Trimmomatic (v.1.2.14), after which assembly of the genome was performed using Unicycler on Galaxy (https://usegalaxy.org/). Reads with a base call quality score of ≥28 and coverage of ≥40× were assembled using shovill v.1.0.9 (https://github.com/tseemann/shovill), and contigs with coverage below 10% of the average genome coverage were excluded from final assemblies. Assembly quality was evaluated using QUAST (v.5.3.0), implemented on the Galaxy platform (https://usegalaxy.org/) (20). We evaluated key metrics, including total genome length, number of contigs, N50 value, and GC content, to verify assembly contiguity and completeness prior to downstream comparative and plasmid-focused analyses (Table S2). The assembled genomes were analyzed for AMR determinants, plasmid replicons, and genomic features. The ResFinder database (updated 30 July 2020) was used to identify AMR genes, with criteria set at 90% identity and 50% coverage. The PointFinder scheme for *Salmonella* spp. (updated August 2019) implemented in staramr v.0.4.0 (https://github.com/phac-nml/staramr?tab=readme-ov-file#mlsttsv) and the PlasmidFinder database were used to detect specific plasmids with a minimum of 90% identity and 60% coverage (https://www.genomicepidemiology.org/services/). Multilocus sequence typing (MLST) was performed to examine the whole-genome phylogeny using the Cano_wgMLST pipeline (21) with the resulting newick file visualized using the ggtree R package.

Given the inherent limitations of short-read sequencing in fully resolving large plasmid structures, the presence of the pESI-like backbone was inferred using a predefined panel of conserved diagnostic markers as previously described in large-scale genomic studies (4, 10). Specifically, isolates were classified as harboring a pESI-like backbone if at least four of the seven diagnostic markers (*pilL*, *sogS*, *trbA*, *ipf*, *ipr2*, *ardA*, and IncFIB(pN55391)) were detected. This threshold was selected as a conservative operational criterion for identifying pESI-like backbone in short-read assemblies, consistent with thresholds applied in large-scale genomic surveillance of pESI-mediated resistance (22).

The genotype-phenotype concordance analysis was performed by integrating the presence of antimicrobial resistance genes and resistance-associated mutations with the corresponding phenotypic susceptibility profiles. The resulting heatmap was generated using the ComplexHeatmap v.2.10.0 package in R (23). BLAST analysis was conducted to compare these markers against the representative reference plasmid sequence, confirming an identity and coverage greater than 99%. To further quantify genetic relatedness, single-nucleotide polymorphisms (SNPs) were identified among the isolates using the BactSNP pipeline (24). A pairwise SNP distance matrix was subsequently generated using the snp-dists package (https://github.com/tseemann/snp-dists). The resulting matrix was visualized as a heatmap using the ComplexHeatmap v.2.10.0 package in R (Fig. S1) (23).

### Comparative plasmid alignment

To contextualize the Lebanese isolates, we conducted a comparative analysis using publicly available complete pESI-like megaplasmid sequences from representative isolates in Turkey, Italy, and the United States. Reference sequences were selected to represent well-characterized pESI/pESI-like plasmids from geographically and epidemiologically relevant poultry-associated lineages, prioritizing publicly available complete or high-quality assemblies suitable for comparative alignment. Specifically, Italy and Turkey were selected to provide Mediterranean/regional context relevant to Lebanon, while the United States was included as a well-characterized North American poultry-associated pESI-positive lineage. The complete pESI-like plasmid sequence CP047882.1 from *S*. Infantis strain 119944 was selected as the reference because it represents a publicly available complete pESI-like plasmid that has been used in previous studies for pESI-like plasmid screening and comparative analyses (22, 25). The 13 most complete plasmid assemblies among our Lebanese isolates, together with three representative isolates from each of Turkey, Italy, and the United States, were aligned against the reference sequence CP047882.1 using the mummer2circos package (26, 27). The nucmer aligner was applied to generate alignment coordinates, which were then visualized with Circos (28). For inter-country variation in AMR gene content, a single Lebanese isolate (SRR17614878) was compared against publicly available genomes from Turkey (S18/0141, S18/0143, and S18/0145), Italy (ERR1014112, ERR1014114, and ERR1014117), and the United States (SRR2407791, SRR2939562, and SRR3184311) (10, 11).

## RESULTS

### Antimicrobial resistance profiles of the 72 *Salmonella* Infantis isolates

AST on the 72 *S*. Infantis isolates revealed significant resistance to commonly used antibiotics (Table S1). Disk diffusion analysis showed high resistance rates against TET (90.3%, *n* = 65) and CIP (36.1%, *n* = 26), with lower resistance observed for AMP (15.3%, *n* = 11), CXI (11.1%, *n* = 8), and CHL (8.3%, *n* = 6). Minimal resistance was detected for GEN (5.5%, *n* = 4) and CTR (1.4%, *n* = 1), while no resistance was observed against MER. Additionally, broth microdilution results confirmed resistance to NAL (100%, *n* = 72) and high resistance to TRS (95.8%, *n* = 69), whereas all isolates were sensitive to AZI (Table 1).

**TABLE 1:** Antimicrobial susceptibility testing results for 72 *Salmonella* Infantis isolates^*a*^

Antibiotic class	Antibiotic agent	Resistant isolates, *n* (%)
Tetracyclines	Tetracycline (TET)	65 (90.3%)
Quinolones	Nalidixic acid (NAL)	72 (100%)
	Ciprofloxacin (CIP)	26 (36.1%)
Sulfonamides	Trimethoprim-sulfamethoxazole (TRS)	69 (95.8%)
β-Lactams	Ampicillin (AMP)	11 (15.3%)
	Cefoxitin (CXI)	8 (11.1%)
	Amoxicillin-clavulanic acid (AMC)	5 (6.9%)
	Ceftriaxone (CTR)	1 (1.4%)
	Meropenem (MER)	0 (0%)
Phenicols	Chloramphenicol (CHL)	6 (8.3%)
Aminoglycosides	Gentamicin (GEN)	4 (5.5%)
Macrolides	Azithromycin (AZI)	0 (0%)

^*a*^ Susceptibility was determined using disk diffusion for tetracycline (TET), ciprofloxacin (CIP), ampicillin (AMP), cefoxitin (CXI), ceftriaxone (CTR), meropenem (MER), chloramphenicol (CHL), and gentamicin (GEN). Broth microdilution was used for nalidixic acid (NAL), trimethoprim-sulfamethoxazole (TRS), amoxicillin-clavulanic acid (AMC), and azithromycin (AZI). Results were interpreted according to CLSI M100 ED34:2024 guidelines, except for CXI, GEN, AMC, and NAL, which were interpreted using EUCAST criteria. Resistance was defined by the following specific cut-off values: nalidixic acid: MIC ≥ 32 µg/mL, trimethoprim-sulfamethoxazole: MIC ≥ 4/76 µg/mL (1:19 ratio), amoxicillin-clavulanic acid: MIC ≥ 32 µg/mL, azithromycin: MIC ≥ 32 µg/mL.

### Genomic features and functional role of the pESI-like plasmid

**Fig 1:**
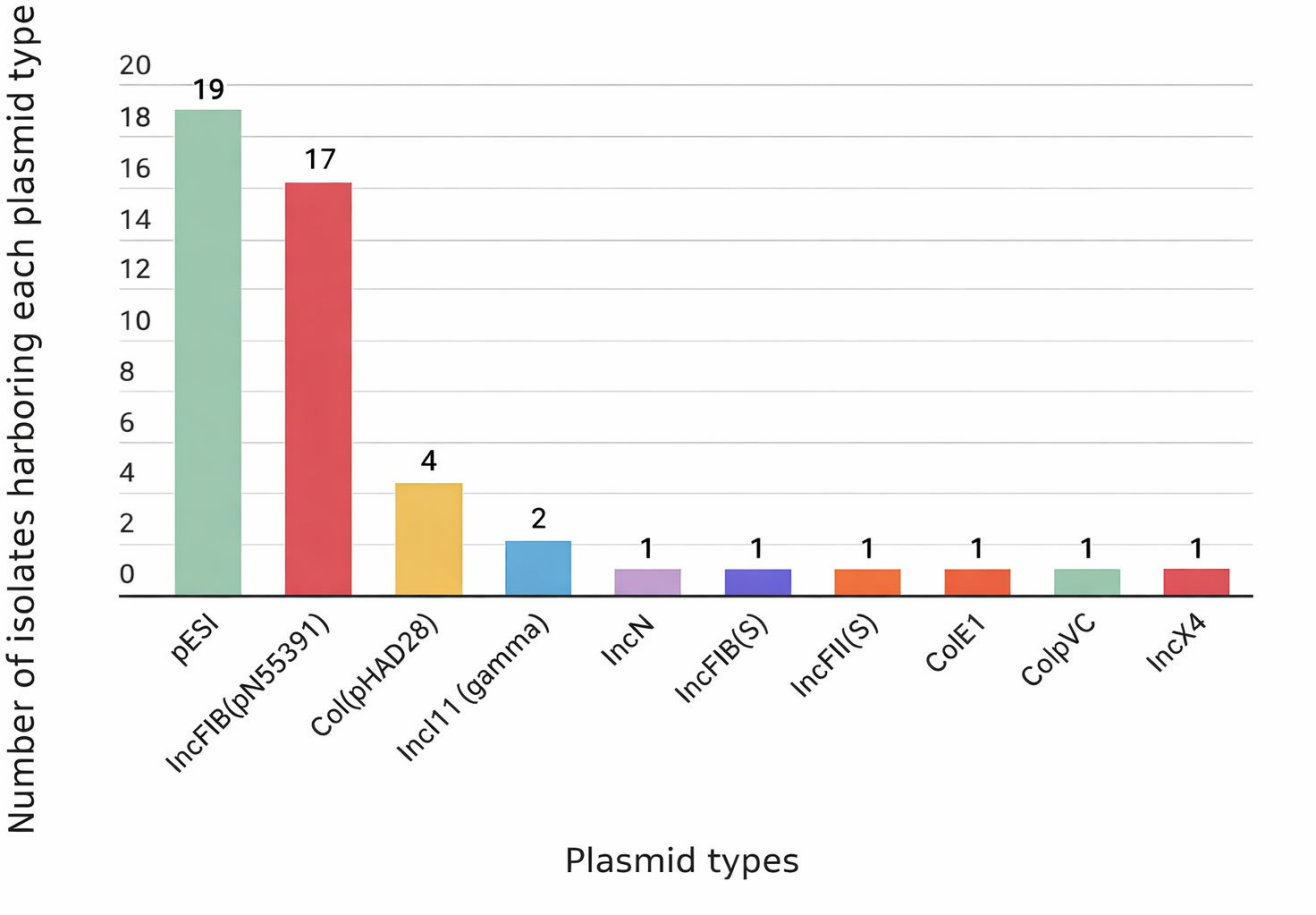
Distribution of plasmids in the 19 sequenced *S*. Infantis isolates. This bar graph illustrates the presence of various plasmids in the 19 *S*. Infantis isolates. pESI-like plasmid markers were detected in all sequenced isolates. IncFIB(pN55391) was detected in 17 *S*. Infantis isolates, Col(pHAD28) was present in four different isolates, and IncI1-I(gamma) was present only in two isolates. Additional replicon types, including IncX1, IncFIB(S), IncFII(S), ColE1, ColpVC, and IncX4, were each found in a single isolate. Bar heights reflect the abundance of each plasmid type across the 19 genomes.

Assembly statistics for the 19 *S*. Infantis genomes are shown in Table S2. Briefly, assemblies consisted of 29–109 contigs (median 60) with total lengths ranging from 4.68 to 5.13 Mb (median 4.97 Mb). N50 values ranged between 106.9 and 694.3 kb (median 206.7 kb), indicating good assembly continuity for Illumina short-read data. GC content was highly consistent across all isolates (52.04%–52.28%, mean 52.12%), as expected for *S*. Infantis. Together, these metrics support the overall high quality of the assemblies used for downstream pESI-like plasmid characterization. Plasmid analysis of the 19 sequenced *S*. Infantis isolates revealed marker profiles consistent with the presence of a pESI-like plasmid backbone in all isolates (Fig. 1). The IncFIB(pN55391) replicon, a key marker of pESI-like plasmids, was identified in 89.5% (*n* = 17) of the isolates, emphasizing its role as a conserved genetic backbone. Additional plasmid types, such as Col(pHAD28) and IncI1-I(gamma), were found in 21% (*n* = 4) and 11% (*n* = 2) of the isolates, reflecting genetic variability. Comparative genomic alignment revealed that the pESI-like backbone was associated with a consistent MDR gene cluster. Assembly fragmentation limited definitive resolution of physical linkage on single contigs across all isolates (Table S2). However, key resistance determinants such as *sul1*, *tet(A*), and *aadA* mapped to corresponding regions of the complete pESI reference sequence CP047882.1 with high identity and coverage, supporting their inferred plasmid-borne association. Definitive plasmid-level localization could not be fully resolved from short-read assemblies.

### Distribution of pESI-like plasmid markers

The presence of pESI-like plasmids among the sequenced *S*. Infantis isolates was evaluated based on a panel of conserved genetic markers (Table 2). The *ardA* gene, encoding a restriction-modification inhibitor, was detected in 100% (*n* = 19) of the isolates, making it the most conserved pESI marker. The plasmid replicon IncP, as well as K88 (fimbrial adhesin) and *trbA* (plasmid transfer regulation), were each found in 94.7% (*n* = 18) of the genomes. Additional core markers—including IncFIB(pN55391), *ipf* (invasion plasmid factor), *sogS* (primase), *ybt* (yersiniabactin biosynthesis), *pilL* (pilLus biosynthesis), and *pESIrepA*—were also widely distributed, each occurring in 89.5% (*n* = 17) of isolates, reflecting their broad distribution and potential functional importance. The mercury resistance gene *merA* was identified in 78.9% (*n* = 15) of isolates, while the conserved pESI hypothetical backbone region was detected in 68.4% (*n* = 13). Collectively, these results support the widespread distribution of pESI-like plasmid backbones and their functional diversity within the Lebanese *S*. Infantis population.

**TABLE 2:** Distribution of pESI-associated genetic markers across sequenced *S*. Infantis isolates^*a*^

Sample SRR	IncFIB_pN55391	IncP	K88	*ardA*	*ipf*	*merA*	pESIrepA	*pilL*	*sogS*	*trbA*	*ybt*	pESI hypothetical backbone
SRR17614876	+	+	+	+	+	+	+	−	+	+	+	−
SRR17614877	+	+	+	+	+	+	+	+	−	+	+	+
SRR17614878	+	+	+	+	+	+	+	+	+	+	+	−
SRR17614879	+	+	+	+	+	+	+	+	+	+	+	+
SRR17614881	+	+	+	+	+	+	+	+	+	+	+	−
SRR17614882	+	+	+	+	+	+	+	+	+	+	+	+
SRR17614883	+	+	+	+	+	+	+	+	+	+	+	+
SRR17614884	+	+	+	+	+	−	+	+	+	+	+	+
SRR17614885	+	+	+	+	+	−	+	+	+	+	+	+
SRR17614886	+	+	+	+	+	+	+	+	−	+	+	+
SRR17614900	+	+	+	+	+	+	+	+	+	−	+	+
SRR17614901	+	+	+	+	+	+	+	+	+	+	+	+
SRR17614902	+	+	+	+	+	+	+	−	+	+	+	−
SRR17614903	+	+	+	+	+	+	+	+	+	+	+	+
SRR17614905	+	+	+	+	+	+	+	+	+	+	+	+
SRR17614906	−	+	−	+	−	−	−	+	+	+	−	−
SRR17614907	−	−	+	+	−	−	−	+	+	+	−	−
SRR8833924	+	+	+	+	+	+	+	+	+	+	+	+
SRR8833925	+	+	+	+	+	+	+	+	+	+	+	+
Total positive	17/19: 89.5%	18/19: 94.7%	18/19: 94.7%	19/19: 100%	17/19: 89.5%	15/19: 78.9%	17/19: 89.5%	17/19: 89.5%	17/19: 89.5%	18/19: 94.7%	17/19: 89.5%	13/19: 68.4%

^*a*^ The table displays the presence (+) or absence (−) of genetic markers associated with the pESI megaplasmid in 19 *Salmonella* Infantis isolates. Markers include plasmid replicons (IncFIB_pN55391, IncP), maintenance and conjugative transfer genes (pESIrepA, *pilL*, *sogS*, and *trbA*), virulence and fitness factors (*ardA*, *ipf*, *ybt*, and K88), and the mercury tolerance gene merA. The final row summarizes the overall prevalence (no. and %) of each marker, demonstrating the high conservation of the pESI backbone and its associated cargo across the surveyed lineage.

### Comparative plasmid analysis of Lebanese and international isolates

**Fig 2:**
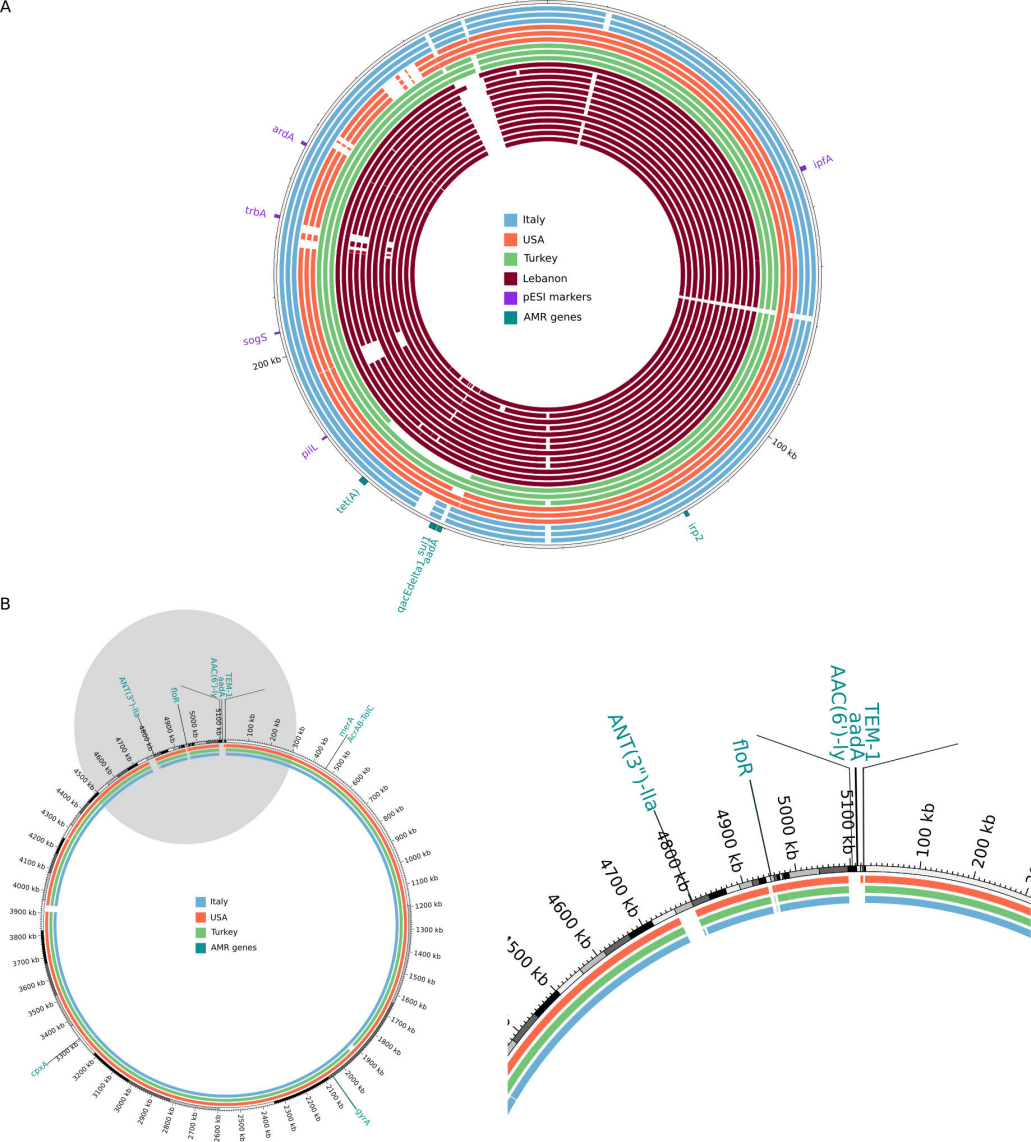
Comparative pESI plasmid alignment and whole-genome AMR gene content among Lebanese and international *Salmonella* Infantis isolates. (**A**) Circular alignment of pESI-like regions from 13 Lebanese *S*. Infantis isolates and representative international isolates from Turkey, Italy, and the United States against the complete pESI plasmid reference CP047882.1 from *S*. Infantis strain 119944. The scale is shown in the figure, and the reference pESI plasmid CP047882.1 is 285,081 bp in length, approximately 285 kb. Because the Lebanese genomes were generated using Illumina short-read sequencing and remain draft assemblies, the aligned Lebanese sequences represent pESI-like regions/contigs mapped to the complete reference plasmid rather than fully closed Lebanese plasmids. (**B**) Whole-genome AMR gene content comparison among selected Lebanese and international *S*. Infantis assemblies. The scale is shown in the figure and corresponds to the full genome/assembly context of approximately 5.1 Mb, not to the size of a pESI plasmid. This panel summarizes the distribution of AMR determinants detected across the analyzed assemblies to highlight inter-country variation in resistance gene content.

Comparative plasmid alignment with representative isolates from Turkey, Italy, and the United States (Fig. 2A) revealed a conserved pESI backbone among the representative plasmids analyzed from these regions. The core pESI backbone, including conjugation-associated loci (*trbA*), fimbrial adhesins (*pilL*, K88), invasion plasmid factor (*ipf*), restriction-modification enzyme (*ardA*), and plasmid maintenance genes (*sogS*), was conserved in nearly all isolates.

To explore genome-level AMR gene variation, we conducted a focused comparison using one Lebanese isolate (SRR17614878) as the reference against representative isolates from Turkey, Italy, and the United States (Fig. 2B). While *tet(A)*, *floR*, and *ANT(3″)-IIa*, among others, were consistently present in isolates from all four countries, other genes such as *blaTEM-1* and *aadA* were geographically restricted. Lebanese isolates aligned more closely with Mediterranean counterparts (Turkey and Italy), reinforcing the regional circulation of pESI-positive *S*. Infantis. Notably, this study constitutes the first genomic evidence of pESI-positive *S*. Infantis in poultry from Lebanon, thereby filling a critical regional gap in global AMR surveillance.

### Phylogenetic relationships and antimicrobial resistance genes

**Fig 3:**
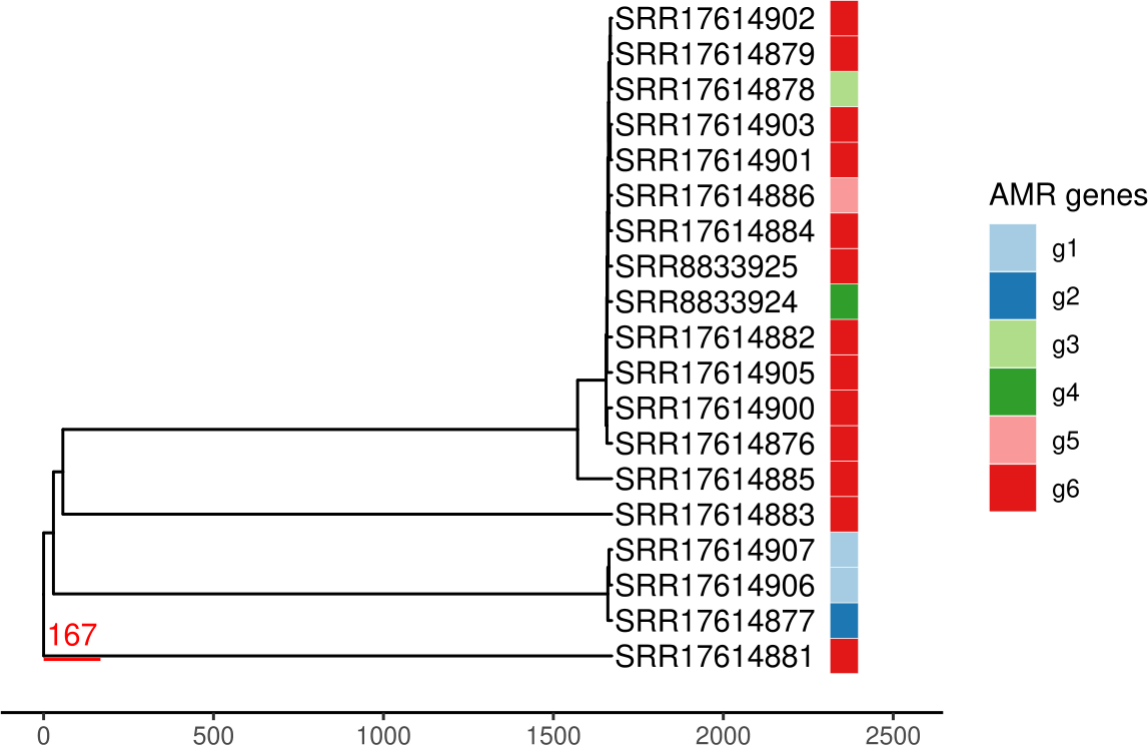
cgMLST-based phylogeny and antimicrobial resistance gene clusters of the 19 Lebanese *S*. Infantis isolates. The rectangular phylogenetic tree shows the core-genome relationships among 19 Lebanese *S*. Infantis isolates based on cgMLST analysis. Branch lengths are proportional to core-genome allelic differences (horizontal scale bar). The red value (167) indicates the maximum core-genome distance separating isolates within the main Lebanese cluster from more distantly related isolates. The colored bar to the right of the tree indicates the AMR gene clusters (g1–g6) defined in this study: g1, *ant(3)-Ia*, *gyrA(D87Y)*, *qacE*, *sul1*, *tet(A*); g2, *ant(3) Ia*, *dfrA1*, *fosA*, *gyrA(D87Y)*, *qacE*, *sul1*, *tet(A*); g3, *aac(3)-Id*, *aadA7*, *aph(6)-Id*, *bla*_CMY-2_, *bla*_TEM1B_, *gyrA(D87N)*, *gyrA(S83F)*, *parC(S80I)*, *qacE*, *sul1*, *tet(A*); g4, *ant(3) Ia*, *gyrA(D87Y)*, *qacE*, *qnrB19*, *sul1*, *tet(A*); g5, *gyrA(D87N*); g6, *ant(3)-Ia*, *bla*_TEM-1B_, *floR*, *gyrA(D87Y)*, *qacE*, *sul1*, and *tet(A*).

Core genome multilocus sequence typing (cgMLST) of the 19 sequences of *S*. Infantis isolates showed that most isolates fell into a single cluster within the sampled Lebanese poultry-associated population (Fig. 3). Within this main cluster, core-genome allelic distances between isolates were ≤167, whereas distances to isolates outside the cluster were substantially higher, supporting the presence of a dominant cluster within the sampled regional poultry population. Pairwise core-genome SNP distances supporting these clusters are provided in Fig. S1. Hierarchical clustering identified six distinct AMR determinant clusters (g1–g6), characterized as follows: g1: *ant(3)-Ia*, *gyrA(D87Y)*, *qacE*, *sul1*, *tet(A*); g2: *ant(3)-Ia*, *dfrA1*, *fosA*, *gyrA(D87Y)*, *qacE*, *sul1*, *tet(A*); g3: *aac(3)-Id*, *aadA7*, *aph(6)-Id*, *bla*_CMY-2_, *bla*_TEM-1B_, *gyrA(D87N)*, *gyrA(S83F)*, *parC(S80I)*, *qacE*, *sul1*, *tet(A*); g4: *ant(3)-Ia*, *gyrA(D87Y)*, *qacE*, *qnrB19*, *sul1*, *tet(A*); g5: *gyrA(D87N*); and g6: *ant(3)-Ia*, *bla*_TEM-1B_, *floR*, *gyrA(D87Y*), *qacE*, *sul1*, and *tet(A*). Genes conferring resistance against tetracyclines *tet(A*) and sulfonamides (*sul1*) were widespread, while fluoroquinolone resistance was linked to mutations in *gyrA*.

**Fig 4:**
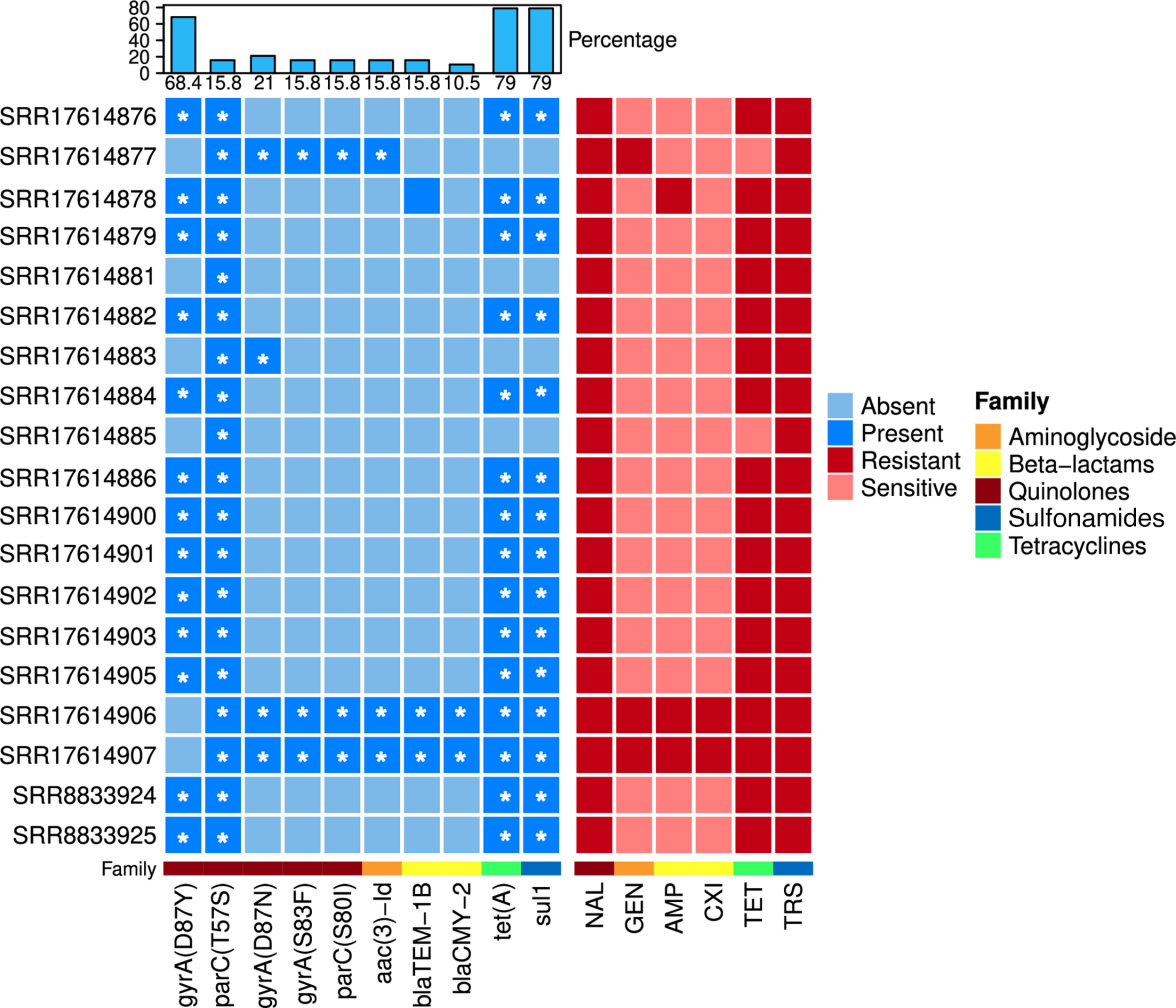
Integrated genotype–phenotype heatmap of antimicrobial resistance among 19 sequenced *S*. Infantis isolates. The heatmap displays the direct comparison between the presence of AMR genes (genotype) and the corresponding phenotypic susceptibility status (phenotype) for each isolate. Columns are grouped by antimicrobial class. Genotype columns indicate the presence (dark color) or absence (light color) of specific resistance determinants. Phenotype columns indicate whether the isolate was resistant (R) or susceptible (S) to the representative antibiotic of that class. White asteriks within genotype cells indicate genotype–phenotype concordance, defined as the presence of an AMR determinant together with resistance to the corresponding antimicrobial agent. The analysis reveals a high degree of concordance between the pESI-like resistome and the observed MDR phenotypes, particularly for tetracyclines, sulfonamides, and quinolones.

Additionally, to validate the functional impact of the identified resistome, we integrated WGS data with phenotypic susceptibility profiles ( Fig. 4 ). The analysis revealed a high degree of concordance between genotype and phenotype across the 19 sequenced isolates. Specifically, 94. 7 % ( *n* = 18 ) of isolates carrying the *tet(A* ) gene exhibited phenotypic resistance to tetracycline. Similarly, 100 % of isolates harboring the *sul1* gene and chromosomal QRDR mutations ( *gyrA* or *parC* ) were phenotypically resistant to trimethoprim-sulfamethoxazole and nalidixic acid, respectively. Concordance was also observed for β-lactams, where resistance to ampicillin and cefoxitin tallied with the presence of *blaTEM-1B* or *blaCMY-2* . Overall, genotype–phenotype concordance was high across the analyzed antimicrobial classes, with only one d iscordant case in which a resistance determinant was detected but the corresponding phenotype remained susceptible. These results support a strong concordance between detected AMR determinants and the corresponding phenotypic resistance profiles among the sequenced Lebanese poultry isolates.

## DISCUSSION

This study provides the first comprehensive genomic characterization of pESI-positive MDR *Salmonella enterica* serovar Infantis from poultry in Lebanon, highlighting poultry production as a potential reservoir for MDR *S*. Infantis at the human–animal–environment interface. The high prevalence of multidrug resistance, particularly to quinolones and tetracyclines, further amplifies the public health relevance of these isolates (29, 30).

Importantly, the widespread detection of pESI-like markers together with AMR determinants supports an association between this megaplasmid-associated lineage and antimicrobial resistance in Lebanese poultry isolates, consistent with reports showing that pESI-like plasmids contribute to persistence, adaptation, and dissemination of MDR *S*. Infantis in poultry systems (4, 31). Moreover, pESI-like plasmids may disseminate through vertical transmission of successful *S*. Infantis lineages and horizontal conjugative transfer to other *Salmonella* serovars or commensal Enterobacterales. Their maintenance in poultry systems may be further favored by antimicrobial, metal, and disinfectant selective pressures (4, 32, 33).

Antimicrobial susceptibility testing revealed that 93% of Lebanese *S*. Infantis isolates were classified as MDR, with high resistance to TET (90.3%, *n* = 65), TRS (96%, *n* = 69), and NAL (100%, *n* = 72). These resistance patterns are consistent with previous reports of high TET and TRS resistance among poultry-associated bacteria in Lebanon and may reflect selective pressures within poultry production systems (3, 17, 34). However, because antimicrobial usage data were not available for the sampled farms, these resistance patterns cannot be directly attributed to specific antimicrobial practices. An alternative, non-mutually exclusive explanation is that *S*. Infantis, potentially through pESI-like plasmid carriage or other adaptive traits, may be well adapted to chickens and poultry-production environments. Moreover, the observed NAL resistance, linked to *gyrA* and *parC* mutations (D87Y, S80I), is also consistent with reports of quinolone resistance and MDR profiles among poultry-associated *S*. Infantis from the Middle East, Europe, and the United States (11, 35–37). Overall, the WGS data showed high concordance with the phenotypic susceptibility profiles, supporting the functional relevance of the detected AMR determinants, including *tet(A*) and *sul1*, as well as chromosomal mutations, in the observed MDR phenotypes.

The detection of β-lactamase genes *bla*_TEM-1B_ and *bla*_CMY-2_, while limited, prompts urgent monitoring to prevent potential spread of resistance against cephalosporins that remain largely effective, such as CTR. In contrast, susceptibility to AZI and MER suggests that these agents remain effective therapeutic options against the tested isolates. The persistence of pESI-positive *S*. Infantis in poultry environments may also be supported by co-selection pressures, including antimicrobial use, heavy metals, and disinfectants, as well as environmental reservoirs such as broiler litter and agricultural surface waters (7, 38–40).

Genomically, the 19 representative MDR *S*. Infantis isolates showed widespread detection of pESI-like markers, including IncFIB(pN55391), *ardA*, *trbA*, and *ipf*, supporting the broad distribution of this megaplasmid-associated marker profile among Lebanese isolates. The diagnostic markers used for pESI-like plasmid identification were selected based on their documented high conservation across international lineages and previous pESI-like surveillance studies (4, 10, 11, 22, 41, 42). Key AMR genes, including *sul1*, *tet(A)*, and *aadA*, were frequently detected among isolates carrying pESI-like markers and were consistent with the observed MDR phenotypes; however, their definitive physical linkage to the pESI-like backbone could not be fully resolved from short-read assemblies. WGS also revealed 10 plasmid types, with IncFIB(pN55391) detected in 89.5% (*n* = 17) of isolates. This pattern may reflect co-selection of IncFIB(pN55391)-associated pESI-like plasmids in poultry environments, consistent with U.S. reports linking these plasmids to MDR *S*. Infantis lineages (43).

Comparative analysis with representative isolates from Turkey, Italy, and the United States (Fig. 2A) placed the Lebanese isolates within a broader pESI-positive *S*. Infantis context. The conserved backbone markers observed in Lebanese isolates are consistent with previous large-scale analyses identifying IncFIB(pN55391) and the *sogS*–*trbA*–*pilL* cluster as common pESI-like backbone elements (10, 11).

Although core pESI-associated markers were conserved, accessory AMR gene content varied across regions (Fig. 2B). Certain resistance determinants, such as *tet(A*), *floR*, and *ANT(3″)-IIa*, were detected across isolates from all four countries, whereas genes such as *blaTEM-1* and *aadA* showed more variable geographic distribution (44). These differences are likely influenced by regional antimicrobial usage practices in livestock production, driving plasmid adaptation in distinct agricultural settings.

Beyond Lebanon, recent Middle Eastern studies from Lebanon, Egypt, Iraq, and the Gulf region have similarly reported a high prevalence of pESI-like *S*. Infantis lineages and MDR *Salmonella* along poultry value chains, including ST2283 isolates carrying IncFIB(pN55391)-type pESI-like plasmids and extended-spectrum β-lactamases such as blaCTX-M-65 (3, 36, 37, 45, 46). Together, these findings place Lebanese poultry isolates within a broader Mediterranean, Middle Eastern, and North American context of pESI-positive MDR *S*. Infantis circulation and provide baseline genomic evidence from an underrepresented region.

The integration of isolates from the Mediterranean basin (Italy and Turkey) and North America into our analysis highlights Lebanon as part of the global network of pESI-positive *S*. Infantis. Importantly, this study provides the first genomic evidence of pESI-positive *S*. Infantis in poultry from Lebanon and fills an important regional surveillance gap.

Phylogenetic analysis (Fig. 3) revealed that most Lebanese isolates fell within a single cluster in the sampled regional poultry population. Despite overall genetic homogeneity, six distinct AMR gene clusters were identified (Fig. 3), demonstrating notable diversity in resistance determinants within the population. Because farm-level metadata were unavailable, this clustering could not be attributed to specific farms or direct farm-to-farm transmission, but may reflect circulation within the broader regional poultry production network (47).

Several limitations warrant consideration. The small WGS sample size (19 isolates) and single-month sampling period (March 2017) provide a cross-sectional snapshot that may limit the generalizability of temporal or seasonal trends. In addition, reliance on short-read sequencing resulted in fragmented assemblies, preventing complete circularization of the pESI-like megaplasmid and definitive confirmation of AMR gene linkage across all isolates (Table S2). Future longitudinal farm-to-fork studies are needed to better define the persistence, turnover, and transmission dynamics of pESI-positive *S*. Infantis lineages (11, 35, 48).

Nevertheless, recent studies from 2024 to 2025 in the Middle East, including Lebanon, the UAE, and Egypt, continue to report a high prevalence of pESI-positive MDR *S*. Infantis with similar resistance profiles to those identified in our 2017 study (36, 37, 45, 46). These consistent findings suggest that the pESI-like lineage remains an ecologically stable and persistent threat in the regional poultry production chain.

Future work in Lebanon and the wider region should be framed explicitly within a One Health paradigm, integrating isolates and metadata from humans, food-producing animals, companion animals, and key environmental compartments such as litter, irrigation water, and wastewater to map transmission pathways and identify intervention points (31, 49). Harmonized regional surveillance, including standardized sampling, antimicrobial susceptibility testing, WGS pipelines, and data sharing, will be important for monitoring pESI-positive *S*. Infantis and evaluating the impact of interventions such as vaccination, biosecurity improvements, and antimicrobial-use policies (50, 51).

In conclusion, this study provides the first genomic evidence of pESI-positive *S*. Infantis in poultry in Lebanon, positioning the country within the global network of MDR strains. Our findings highlight the conserved yet adaptable nature of the pESI megaplasmid and emphasize the urgent need for strengthened One Health surveillance, antimicrobial stewardship, and biosecurity interventions in Lebanon and the wider Eastern Mediterranean region.

## Supplementary Material

Reviewer comments

## Data Availability

Raw Illumina sequencing reads for the 19 Lebanese isolates analyzed in this study are available in the NCBI Sequence Read Archive under BioProject accession number PRJNA293224. The sequencing runs associated with these samples are SRR17614876, SRR17614877, SRR17614878, SRR17614879, SRR17614881, SRR17614882, SRR17614883, SRR17614884, SRR17614885, SRR17614886, SRR17614900, SRR17614901, SRR17614902, SRR17614903, SRR17614905, SRR17614906, SRR17614907, SRR8833924, and SRR8833925.

## SUPPLEMENTAL MATERIAL

Tables S1 and S2, and Figure S1 spectrum.02418-25-s0001.docx Table S1: Antimicrobial susceptibility profiles of *S. Infantis* isolates based on disk diffusion and broth microdilution methods. Table S2: Assembly statistics for the 19 *S. Infantis* genomes. Figure S1: Pairwise core-genome SNP distances among 19 Lebanese *S. Infantis* isolates.

OPEN PEER REVIEW reviewer-comments.pdf An accounting of the reviewer comments and feedback.

ASM does not own the copyrights to Supplemental Material that may be linked to, or accessed through, an article. The authors have granted ASM a non-exclusive, world-wide license to publish the Supplemental Material files. Please contact the corresponding author directly for reuse.
