# Supplementary material for: Molecular characterization of pESI-like megaplasmids in Salmonella Infantis from poultry in Lebanon
Source: Microbiol Spectr. 2026 Jul 6;14(8):e02418-25. doi: 10.1128/spectrum.02418-25 (PMC13435791; doi:10.1128/spectrum.02418-25)
Supplement: Supplementary file 1 [file reviewer-comments.pdf]

AMERICAN SOCIETY FOR MICROBIOLOGY | Microbiology Spectrum

Molecular Characterization of pESI- like Megaplasmids in Salmonella Infantis from Poultry in Lebanon

Sara Barada, Jose Rita Gerges, Hadi Hussein, Abdallah Kurdi, Antoine Abou Fayad, Rima El hage, and Ghassan Matar

Corresponding Author(s): Ghassan Matar, American University of Beirut

Review Timeline:

Submission Date:	August 25, 2025
Editorial Decision:	November 19, 2025
Revision Received:	December 15, 2025
Editorial Decision:	January 11, 2026
Revision Received:	January 29, 2026
Editorial Decision:	February 16, 2026
Revision Received:	March 29, 2026
Editorial Decision:	April 27, 2026
Revision Received:	May 22, 2026
Accepted:	June 1, 2026

Editor: John Osei Sekyere

Reviewer(s): Disclosure of reviewer identity is with reference to reviewer comments included in decision letter(s). The following individuals involved in review of your submission have agreed to reveal their identity: Bui Phuc Bui Hoang Phuc (Reviewer #2); Yichen Ding (Reviewer #4)

Transaction Report:

(Note: With the exception of the correction of typographical or spelling errors that could be a source of ambiguity, letters and reports are not edited. The original formatting of letters and referee reports may not be reflected in this compilation.)

DOI: https://doi.org/10.1128/spectrum.02418-25

Re: Spectrum02418-25 (Molecular Characterization of pESI- like Megaplasmids in Salmonella Infantis from Poultry in Lebanon)

Dear Dr. Ghassan M Matar:

Thank you for the privilege of reviewing your work. Below you will find my comments, instructions from the Spectrum editorial office, and the reviewer comments.

Please return the manuscript within 60 days; if you cannot complete the modification within this time period, please contact me. If you do not wish to modify the manuscript and prefer to submit it to another journal, notify me immediately so that the manuscript may be formally withdrawn from consideration by Spectrum.

Revision Guidelines
To submit your modified manuscript, log into the submission site at https://spectrum.msubmit.net/cgi-bin/main.plex. Go to Author Tasks and click the appropriate manuscript title to begin. The information you entered when you first submitted the paper will be displayed; update this as necessary. Note the following requirements:

• Upload point-by-point responses to the issues raised by the reviewers in a file named "Response to Reviewers," NOT in your cover letter.
• Upload a compare copy of the manuscript (without figures) as a "Marked-Up Manuscript" file.
• Upload a clean .DOC/.DOCX version of the revised manuscript and remove the previous version.
• Each figure must be uploaded as a separate, editable, high-resolution file (TIFF or EPS preferred), and any multipanel figures must be assembled into one file.
• Any supplemental material intended for posting by ASM should be uploaded with their legends separate from the main manuscript. You can combine all supplemental material into one file (preferred) or split it into a maximum of 10 files with all associated legends included.

For complete guidelines on revision requirements, see our Submission and Review Process webpage. Submission of a paper that does not conform to guidelines may delay acceptance of your manuscript.

Data availability: ASM policy requires that data be available to the public upon online posting of the article, so please verify all links to sequence records, if present, and make sure that each number retrieves the full record of the data. If a new accession number is not linked or a link is broken, provide Spectrum production staff with the correct URL for the record. If the accession numbers for new data are not publicly accessible before the expected online posting of the article, publication may be delayed; please contact production staff (Spectrum@asmusa.org) immediately with the expected release date.

Publication Fees: For information on publication fees and which article types are subject to charges, visit our website. If your manuscript is accepted for publication and any fees apply, you will be contacted separately about payment during the production process; please follow the instructions in that e-mail. Arrangements for payment must be made before your article is published.

ASM Membership: Corresponding authors may join or renew ASM membership to obtain discounts on publication fees. Need to upgrade your membership level? Please contact Customer Service at Service@asmusa.org.

The ASM Journals program strives for constant improvement in our submission and publication process. Please tell us how we can improve your experience by taking this quick Author Survey.

Thank you for submitting your paper to Spectrum.

Sincerely,
John Osei Sekyere
Editor
Microbiology Spectrum

Reviewer #1 (Comments for the Author):

Thanks for addressing review comments made to the previous version. The manuscript has been improved, which can provide some useful information to the readers.

Still, please re-consider the ranking of tetracyclines to be revised from "critically important" to "highly important", according to

WHO's ranking. Perhaps, only newer compound in the class, such as doxycycline may be ranked higher.

Reviewer #2 (Comments for the Author):

This manuscript represents an important and novel contribution, being the first genomic characterization of pESI-positive S. Infantis isolates from Lebanon and filling a critical gap in AMR surveillance in the Middle East. The study is methodologically rigorous, combining phenotypic AST with WGS, and the findings have clear public health relevance. However, several minor revisions are required before publication:
(1) In the Abstract, please provide quantitative data on MDR prevalence (e.g., "93% [67/72] of isolates were MDR") to strengthen the summary.
(2) In the Introduction, highlight Lebanon's strategic geographic position as a sentinel site for AMR spread across continents.
(3) In Materials & Methods, clarify the criteria for selecting the 19 isolates for WGS, and
(4) add assembly quality metrics (N50 values, genome size range) to ensure transparency.
(5) In the Results, improve Table 2 by adding a final row with overall prevalence percentages, and
(6) explicitly connect fluoroquinolone resistance mutations (gyrA/parC) to the observed phenotypic resistance.
(7) In the Discussion, expand on mechanisms of pESI dissemination (vertical transmission, horizontal transfer, selective pressure, environmental contamination), and (8) compare findings with other Middle Eastern studies to situate the work in a broader regional context.
(9) Provide a more detailed outlook on future research priorities, including longitudinal surveillance, One Health approaches, and regional collaboration.
(10) Correct minor typographical and nomenclature errors (e.g., "S801" → "S80I," "Unites States" → "United States"), and (11) revise figure legends for clarity and consistency.
(12) Optionally, consider adding a supplementary conceptual figure illustrating pESI structure, transmission, and genotype-phenotype links to aid non-specialist readers.

This manuscript represents an important and novel contribution, being the first genomic characterization of pESI-positive S. Infantis isolates from Lebanon and filling a critical gap in AMR surveillance in the Middle East. The study is methodologically rigorous, combining phenotypic AST with WGS, and the findings have clear public health relevance. However, several minor revisions are required before publication:
(1) In the Abstract, please provide quantitative data on MDR prevalence (e.g., “93% [67/72] of isolates were MDR”) to strengthen the summary.
(2) In the Introduction, highlight Lebanon’s strategic geographic position as a sentinel site for AMR spread across continents.
(3) In Materials & Methods, clarify the criteria for selecting the 19 isolates for WGS, and
(4) add assembly quality metrics (N50 values, genome size range) to ensure transparency.
(5) In the Results, improve Table 2 by adding a final row with overall prevalence percentages, and
(6) explicitly connect fluoroquinolone resistance mutations (gyrA/parC) to the observed phenotypic resistance.
(7) In the Discussion, expand on mechanisms of pESI dissemination (vertical transmission, horizontal transfer, selective pressure, environmental contamination), and (8) compare findings with other Middle Eastern studies to situate the work in a broader regional context.
(9) Provide a more detailed outlook on future research priorities, including longitudinal surveillance, One Health approaches, and regional collaboration.
(10) Correct minor typographical and nomenclature errors (e.g., “S801” → “S80I,” “Unites States” → “United States”), and (11) revise figure legends for clarity and consistency.
(12) Optionally, consider adding a supplementary conceptual figure illustrating pESI structure, transmission, and genotype–phenotype links to aid non-specialist readers.

Thanks for addressing review comments made to the previous version. The manuscript has been improved, which can provide some useful information to the readers.

Reviewer #1 (Comments for the Author):

Still, please re-consider the ranking of tetracyclines to be revised from "critically important" to "highly important", according to WHO's ranking. Perhaps, only newer compound in the class, such as doxycycline may be ranked higher.

- The "critically important" ranking of tetracyclines has been revised to "highly important" throughout the manuscript as suggested.

Reviewer #2 (Comments for the Author):

This manuscript represents an important and novel contribution, being the first genomic characterization of pESI-positive *S.* Infantis isolates from Lebanon and filling a critical gap in AMR surveillance in the Middle East. The study is methodologically rigorous, combining phenotypic AST with WGS, and the findings have clear public health relevance. However, several minor revisions are required before publication:

(1) In the Abstract, please provide quantitative data on MDR prevalence (e.g., "93% [67/72] of isolates were MDR") to strengthen the summary.

- The proportion and percentage were added to the abstract. The sentence now reads: "**A total of 72 isolates were recovered during a nationwide surveillance effort, among which 67 (93%) were MDR based on antimicrobial susceptibility testing (disk diffusion and broth microdilution) results, including resistance to critically important agents such as quinolones, and highly important classes such as tetracyclines and sulfonamides.**"

(2) In the Introduction, highlight Lebanon's strategic geographic position as a sentinel site for AMR spread across continents.

- The following sentence has been added: "**Lebanon occupies a strategic geographic position at the intersection of Asia, Africa and Europe and acts as a regional hub for trade and population mobility making it an important sentinel setting for monitoring the emergence and spread of AMR across continents.**"

(3) In Materials & Methods, clarify the criteria for selecting the 19 isolates for WGS, and

- In the "Whole-genome sequencing section" of "Materials and Methods", the word "randomly" was added to clarify how these 19 isolates were selected, the sentence now reads: "**Whole-genome sequencing (WGS) was performed on 19 MDR S. Infantis**

isolates, randomly selected to represent the genetic diversity of the 72 isolates based on phenotypic and genotypic characteristics."

(4) add assembly quality metrics (N50 values, genome size range) to ensure transparency.

Assembly quality has now been explicitly documented. In the Bioinformatics analysis subsection of the Materials and Methods, we added a description of the QUAST analysis used to obtain assembly statistics for the 19 *S.* Infantis genomes, and we now refer to a new Supplementary Table S2 that reports, for each isolate, the number of contigs, total assembly length, N50, N75, N90, longest and shortest contigs, average contig length, GC content, AuN, and I50: "**Assembly quality was evaluated using QUAST (v. 5.3.0) implemented on the Galaxy platform (https://usegalaxy.org/) (15). We evaluated key metrics including total genome length, number of contigs, N50 value, and GC content to verify assembly contiguity and completeness prior to downstream comparative and plasmid- focused analyses (Table S2)".**

In the Results section (Genomic Features and Functional Role of the pESI-like Plasmid), we also added a brief summary of the main assembly metrics (contig counts, genome size range, N50 range, and GC content), highlighting that all genomes show appropriate size and continuity for downstream comparative and plasmid-focused analyses: "**Assembly statistics for the 19 *S.* Infantis genomes are shown in Supplementary Table S2. Briefly, assemblies consisted of 29–109 contigs (median 60) with total lengths ranging from 4.68 to 5.13 Mb (median 4.97 Mb). N50 values ranged between 106.9 and 694.3 kb (median 206.7 kb), indicating good assembly continuity for Illumina short-read data. GC content was highly consistent across all isolates (52.04–52.28%, mean 52.12%), as expected for *S.* Infantis. Together, these metrics support the overall high quality of the assemblies used for downstream pESI-like plasmid characterization."**

(5) In the Results, improve Table 2 by adding a final row with overall prevalence percentages.

Table 2 has been updated to include a final row summarizing, for each pESI marker, the numbers and the percentage of positive isolates among the 19 sequenced genomes. Specifically, *ardA* was detected in 19/19 isolates (100.0%), *IncP*, *K88*, and *trbA* in 18/19 isolates (94.7%), IncFIB(pN55391), *ipf,* pESIrepA, *pilL*, *sogS*, and *ybt* in 17/19 isolates (89.5%), *merA* in 15/19 isolates (78.9%), and the pESI hypothetical backbone in 13/19 isolates (68.4%). This new summary row makes it easier for readers to appreciate the overall prevalence of each marker.

(6) explicitly connect fluoroquinolone resistance mutations (gyrA/parC) to the observed phenotypic resistance.

- A more explicit link was established between the mutations and the observed phenotypic resistance in the results section. The sentence now reads: **"Four fluoroquinolone**

resistance-associated mutations were identified in the core genes gyrA and parC: gyrA (D87Y), gyrA (D87N), gyrA (S83F), and parC (S80I). These QRDR mutations, present in 68.4%, 21%, 15.8% and 15.8% of the isolates, respectively, are well known to confer nalidixic acid resistance and reduced susceptibility or resistance to fluoroquinolones in *Salmonella* and are consistent with the high phenotypic resistance to nalidixic acid and lower rate of resistance against ciprofloxacin observed in our isolates."

(7) In the Discussion, expand on mechanisms of pESI dissemination (vertical transmission, horizontal transfer, selective pressure, environmental contamination), and (8) compare findings with other Middle Eastern studies to situate the work in a broader regional context. (9) Provide a more detailed outlook on future research priorities, including longitudinal surveillance, One Health approaches, and regional collaboration.

The discussion was modified based on suggestions in comments 7, 8 and 9. The following paragraphs were added:

- **"Current evidence indicates that pESI-like plasmids disseminate through a combination of vertical transmission of successful *S.* Infantis lineages along the poultry production pyramid and horizontal conjugative transfer to other *Salmonella* serovars and commensal Enterobacterales in the gut, with antimicrobial use, metal contamination, and disinfectant exposure providing strong selective pressure that favors their maintenance (23,25,26)."**
- **"Together with the frequent co-location of resistance, metal, and biocide tolerance genes on pESI-like plasmids, these findings support a scenario of co-selection whereby antimicrobial use, heavy metals, and disinfectants in poultry houses jointly maintain MDR S. Infantis clones even when antibiotic exposure is intermittent(29,30)."**
- **"Environmental studies further indicate that pESI-positive S. Infantis and related plasmids can persist in broiler litter and surface waters surrounding agricultural fields, providing additional reservoirs that may re-introduce resistant strains into the production chain(31,32)."**
- **"Beyond Lebanon, recent Middle Eastern studies from Lebanon, Egypt, Iraq, and the Gulf region have reported a high prevalence of *S.* Infantis and MDR *Salmonella* along poultry value chains, with several genomic investigations from the United Arab Emirates and neighboring countries describing ST2283 *S.* Infantis carrying IncFIB(pN55391)-type pESI-like plasmids and extended-spectrum beta-lactamases**

such as blaCTX-M-65 (3,39–42). Taken together, these data indicate that the epidemiological pattern observed in Lebanese poultry, characterized by pESI-positive MDR *S.* Infantis with high fluoroquinolone and tetracycline resistance, is representative of a broader regional trend across the Eastern Mediterranean."

- **"Although this study provides valuable insights into the AMR profiles and genomic characteristics of *S.* Infantis in Lebanon, some limitations warrant consideration. The small WGS sample size (19 isolates) may limit the generalizability of our findings, underscoring the need for longitudinal studies to capture temporal trends. In particular, multi-year, farm-to-fork sampling that repeatedly surveys breeder flocks, broiler farms, slaughterhouses, retail products, and human salmonellosis cases would allow quantification of the persistence, turnover, and seasonal dynamics of pESI-positive *S.* Infantis lineages (37,44)."**
- **"Future work in Lebanon and the wider region should be framed explicitly within a One Health paradigm, integrating isolates and metadata from humans, food-producing animals, companion animals, and key environmental compartments such as litter, irrigation water, and wastewater to map transmission pathways and identify intervention points (24,45). Establishing regional consortia that harmonize sampling strategies, antimicrobial susceptibility testing, and WGS analysis pipelines, and that share data through international platforms, has been highlighted as a critical research and policy priority for AMR and *Salmonella* control globally and would be particularly impactful for Eastern Mediterranean countries(46,47). Within such collaborative frameworks, priority should be given to coordinated longitudinal surveillance of pESI-positive *S.* Infantis, evaluation of interventions such as vaccination and biosecurity upgrades, and assessment of how changes in antimicrobial usage policies influence the prevalence and genomic evolution of MDR clones in the region."**

(10) Correct minor typographical and nomenclature errors (e.g., "S801" → "S80I," "Unites States" → "United States").

Indicated errors have been amended.

(11) Revise figure legends for clarity and consistency.

All figure legends have been revised for clarity and consistency. The updated legends now (i) specify the number of isolates included in each figure, (ii) define all abbreviations and colour codes, and (iii) provide parallel, structured descriptions for multi-panel figures. The revised legends for Figures 1–5 have been updated in the main text as follows:

- **"Figure 1. Rates of antimicrobial resistance exhibited by the 72 *S*. Infantis isolates against selected antimicrobial agents determined through disk diffusion. The bar graph represents the percentage and number of *Salmonella* Infantis isolates (n = 72) exhibiting resistance to various antibiotics determined through disk diffusion. Tetracycline (TET) resistance was the most prevalent (90%), followed by ciprofloxacin (CIP;36%). Resistance to other antibiotics, including ampicillin (AMP), cefoxitin (CXI), chloramphenicol (CHL), gentamicin (GEN), and ceftriaxone (CTR) was less frequent, while no resistance to meropenem (MER) was observed."**

- **"Figure 2. Distribution of plasmids in the 19 sequenced *S.* Infantis isolates. This bar graph illustrates the presence of various plasmids in the 19 *S*. Infantis isolates. pESI-like plasmid was present in all sequenced isolates. IncFIB (pN55391) was detected in 15 *S*. Infantis isolates, Col(pHAD28) was present in 4 different isolates, and IncI1-I (gamma) was present only in two isolates. Additional replicon types, including IncX1, IncFIB(S), IncFII(S), ColE1, ColpVC, and IncX4 were each found in a single isolate. Bar heights reflect the abundance of each plasmid type across the 19 genomes."**

- **"Figure 3. Comparative genomic analysis of pESI-like megaplasmids from studied Lebanese *Salmonella* Infantis isolates, aligned against representative plasmid sequences from three previous studies (Turkey, Italy, and the USA) and the reference pESI sequence CP047882.1.**
  The circos plot depicts the genomic organization of pESI-like megaplasmids from 13 Lebanese *S*. Infantis isolates (red), three isolates from Turkey (green), three isolates from Italy (blue), and three isolates from the United States (orange) against the reference pESI sequence CP047882.1 (white outer ring). Annotated key genetic features are highlighted on the outermost ring, including virulence-associated genes (*ardA*, *ipfA*, *sogS*, *pilL*, *trbA*) and antimicrobial resistance genes (*tet(A), aadA, sul1*).
  Comparative analysis of AMR gene content across selected pESI-like plasmids representing Lebanon, Turkey, Italy and the United States. A single Lebanese isolate (SRR17614878) was selected as the reference to highlight inter-country variation in resistance gene content. Certain resistance determinants such as *tet(A), floR* and *ANT (3")-IIa* were conserved across isolates, notable differences were observed in accessory AMR genes."

- **"Figure 4. cgMLST- based phylogeny, AMR gene profiles, and pESI-like status of the 19 *S.* Infantis isolates. A core genome multilocus sequence typing (cgMLST) phylogenetic tree displays the degree of relatedness among 19 *S*. Infantis isolates. The inner ring indicates the source of isolation (poultry in magenta), while the middle ring represents the AMR gene groups (g1–g6): g1, *ant(3)-Ia, gyrA(D87Y),***

***qacE, sul1, tet(A)*; g2, *ant(3) Ia, dfrA1, fosA, gyrA(D87Y), qacE,sul1, tet(A)*; g3, *aac(3)-Id, aadA7, aph(6)-Id,bla*$_{CMY-2}$*, bla*$_{TEM1B}$*,gyrA(D87N), gyrA(S83F), parC(S80I), qacE, sul1,tet(A)*; g4, *ant(3) Ia,gyrA(D87Y), qacE, qnrB19, sul1, tet(A)*; g5, *gyrA(D87N)*; g6, *ant(3)-Ia, bla*$_{TEM-1B}$*, floR, gyrA(D87Y), qacE, sul1,* and *tet(A*). The outer ring highlights the presence (purple) or absence (white) of the pESI-like megaplasmid."**

- **"Figure 5. Heatmap depicting the distribution and frequency of AMR determinants among sequenced *S.* Infantis isolates. The heatmap illustrates the presence (dark blue) or absence (light blue) of AMR genes across *S*. Infantis isolates. Rows represent specific isolates, and columns correspond to resistance genes grouped by antimicrobial classes to which they confer resistance, including aminoglycosides, fluoroquinolones, disinfectants, sulfonamides, tetracyclines, β-lactams, phenicols, quinolones, trimethoprim, and fosfomycin. Numerical values above the columns indicate the overall prevalence (%) of each gene across the dataset."**

(12) Optionally, consider adding a supplementary conceptual figure illustrating pESI structure, transmission, and genotype-phenotype links to aid non-specialist readers.

We appreciate this thoughtful suggestion and agree that conceptual summaries can be valuable for non-specialist readers. At this stage, however, we opted not to add an additional schematic figure in order to maintain the focus on data-driven visualizations (Figures 1–5), which already integrate pESI structure, dissemination patterns, and genotype–phenotype relationships. Specifically, Figure 3 summarizes the modular organization and international conservation of the pESI-like backbone, Figure 4 links cgMLST-defined lineages with distinct AMR gene clusters and pESI-like status, and Figure 5 provides a comprehensive overview of AMR determinants and their prevalence. In parallel, we have expanded the Introduction and Discussion to more explicitly describe (i) the structural features and adaptive functions of pESI-like plasmids and (ii) mechanisms of dissemination, co-selection, and regional spread. We hope that these combined textual and graphical elements sufficiently guide non-specialist readers without adding a separate conceptual figure.

Re: Spectrum02418-25R1 (Molecular Characterization of pESI- like Megaplasmids in Salmonella Infantis from Poultry in Lebanon)

Dear Dr. Ghassan M Matar:

Thank you for the privilege of reviewing your work. Below you will find my comments, instructions from the Spectrum editorial office, and the reviewer comments.

Please return the manuscript within 60 days; if you cannot complete the modification within this time period, please contact me. If you do not wish to modify the manuscript and prefer to submit it to another journal, notify me immediately so that the manuscript may be formally withdrawn from consideration by Spectrum.

Revision Guidelines
To submit your modified manuscript, log into the submission site at https://spectrum.msubmit.net/cgi-bin/main.plex. Go to Author Tasks and click the appropriate manuscript title to begin. The information you entered when you first submitted the paper will be displayed; update this as necessary. Note the following requirements:

• Upload point-by-point responses to the issues raised by the reviewers in a file named "Response to Reviewers," NOT in your cover letter.
• Upload a compare copy of the manuscript (without figures) as a "Marked-Up Manuscript" file.
• Upload a clean .DOC/.DOCX version of the revised manuscript and remove the previous version.
• Each figure must be uploaded as a separate, editable, high-resolution file (TIFF or EPS preferred), and any multipanel figures must be assembled into one file.
• Any supplemental material intended for posting by ASM should be uploaded with their legends separate from the main manuscript. You can combine all supplemental material into one file (preferred) or split it into a maximum of 10 files with all associated legends included.

For complete guidelines on revision requirements, see our Submission and Review Process webpage. Submission of a paper that does not conform to guidelines may delay acceptance of your manuscript.

Data availability: ASM policy requires that data be available to the public upon online posting of the article, so please verify all links to sequence records, if present, and make sure that each number retrieves the full record of the data. If a new accession number is not linked or a link is broken, provide Spectrum production staff with the correct URL for the record. If the accession numbers for new data are not publicly accessible before the expected online posting of the article, publication may be delayed; please contact production staff (Spectrum@asmusa.org) immediately with the expected release date.

Publication Fees: For information on publication fees and which article types are subject to charges, visit our website. If your manuscript is accepted for publication and any fees apply, you will be contacted separately about payment during the production process; please follow the instructions in that e-mail. Arrangements for payment must be made before your article is published.

ASM Membership: Corresponding authors may join or renew ASM membership to obtain discounts on publication fees. Need to upgrade your membership level? Please contact Customer Service at Service@asmusa.org.

The ASM Journals program strives for constant improvement in our submission and publication process. Please tell us how we can improve your experience by taking this quick Author Survey.

Thank you for submitting your paper to Spectrum.

Sincerely,
John Osei Sekyere
Editor
Microbiology Spectrum

Reviewer #1 (Comments for the Author):

Thanks for modifying the ranking of tetracylcines.

Reviewer #4 (Public repository details (Required)):

The data is deposited in Genbank with accession numbers provided in the manuscript.

Reviewer #4 (Comments for the Author):

1. Figure 1 and Table 1 can be merged into a single figure/table for concise presentation of antibiotic susceptibility results. While the exact assays and cut-off values which were used to determine the susceptibility for each antibiotic should be presented in the figure legend and/or Method.

2. In table 1, row 2 column 3, what is "{greater than or equal to}4/76"?

3. line 185 - 191, number of isolates should be shown together with rate, i.e. TET (90%, n=63). This applies to the entire manuscript.

4. line 156 - 159, Table 2 and Figure 2: The authors defined that pESI-like plasmid is present in the isolate if "plasmid-specific markers, including piL, sogS, trbA, ipf, ipr2, ardA, and IncFIB (pN55391), with at least four of these markers needed to confirm the presence of the pESI-like plasmid". Using this definition, the authors therefore stated that all 19 isolates harbor pESI-like plasmid in Figure 2. Why the authors used this definition? Is it based on previous studies? Any reference? In this study, all isolates were sequenced by Illumina short read and genomes were assembled into draft genomoe level, which in nature limited its usage in detecting large pESI-like plamids with a size of ~280kb. Therefore a more stringent comparative sequence analysis and standard is required to determine if the isolates harbor pESI-like plasmid.

5. line 247 - 249: The conservativity of pESI marker should be determined by analysing all publically available complete pESI-like plasmid sequences, but not only by 19 isolates sequenced in this study.

6. Figure 3A: 13 S. Infantis isolates in this study were compared with international references. If all 19 isolates were determined to have pESI as shown in Figure 2, why only 13 were used for comparison? It will be more convincing if all the 19 isolates harbor a relative integral plasmid backbone as the 13 isolates shown in Figure 3A to conclude that all 19 isolates harbour pESI-like plasmid.

7. Figure 4: if all isolates had the same source of isolation, the source should not be shown in the figure to avoid redundancy.

8. line 301 - 303: How did the authors define the isolates have "clear phylogenetic relatedness" based on the cgMLST tree? There is no scale bar for the tree in Figure 4, nor did the authors show the snp distance of the isolates, included any reference genomes. The authors need to provide such information to make this statement.

9. Figure 5: This Figure mis-presented the information. The reviwer suggests the authors to present the following in the figure: the heatmap should consists of 1. presence of AMR gene in the isolate; and 2. if the isolate is resistant or sensitive to the antiboitic of the class. this is to discuss if WGS results tally with phenotypic results.

Thanks for addressing review comments made to the previous version. The manuscript has been improved, which can provide some useful information to the readers.

Reviewer #4 (Comments for the Author):

1. Figure 1 and Table 1 can be merged into a single figure/table for concise presentation of antibiotic susceptibility results. While the exact assays and cut-off values which were used to determine the susceptibility for each antibiotic should be presented in the figure legend and/or Method: We have removed the original Figure 1 and integrated the disk diffusion results with the broth microdilution data into a new, comprehensive Table 1. Furthermore, as requested, we have added detailed information regarding the specific assays (disk diffusion vs. broth microdilution) and the exact resistance breakpoints/cut-off values used. This information has been included in two locations:

- **The Materials and Methods section (Subsection: Antimicrobial Susceptibility Testing): Antimicrobial susceptibility testing was performed using disk diffusion and broth microdilution, following the Clinical & Laboratory Standards Institute (CLSI) M100 ED34:2024 guidelines (13). However, since CLSI did not provide breakpoints for cefoxitin, gentamicin, amoxicillin-clavulanic acid, and nalidixic acid, we referred to the European Committee on Antimicrobial Susceptibility Testing (EUCAST) for these antibiotics (14). Quality control was ensured using Escherichia coli ATCC® 25922 as the reference strain for both testing methods, in accordance with CLSI and EUCAST guidelines. For the disk diffusion method, eight antimicrobials were tested: ceftriaxone CTR (30 µg), cefoxitin CXI (30 µg), meropenem MER (10 µg), ampicillin AMP (10 µg), chloramphenicol CHL (30 µg), gentamicin GEN (10 µg), ciprofloxacin CIP (5 µg) and tetracycline TET (30 µg). Plates were then incubated at 37 °C for 18-24 hours after which the diameters of the inhibition zones were measured and interpreted using standard CLSI and EUCAST breakpoints. Further antimicrobial testing was conducted using the broth microdilution method against four additional antibiotics: trimethoprim-sulfamethoxazole (TRS), amoxicillin-clavulanic acid (AMC), azithromycin (AZI) and nalidixic acid (NAL). Two-fold serial dilutions were performed, with concentrations ranging from 2048 µg/mL to 0.5 µg/mL and incubated at 37 °C for 18-24 hours to determine minimum inhibitory concentrations (MICs) in duplicate. Specific resistance breakpoints for broth microdilution assays were defined as: nalidixic acid (MIC ≥32 µg/mL), trimethoprim-sulfamethoxazole (MIC ≥4/76 µg/mL), amoxicillin-clavulanic acid (MIC ≥32 µg/mL), and azithromycin (MIC ≥32 µg/mL). Isolates that exhibited resistance against three or more antimicrobial classes through either method were classified as MDR.**

- **The Legend of Table 1: Susceptibility was determined using disk diffusion for tetracycline (TET), ciprofloxacin (CIP), ampicillin (AMP), cefoxitin (CXI),**

ceftriaxone (CTR), meropenem (MER), chloramphenicol (CHL), and gentamicin (GEN). Broth microdilution was used for nalidixic acid (NAL), trimethoprim-sulfamethoxazole (TRS), amoxicillin-clavulanic acid (AMC), and azithromycin (AZI). Results were interpreted according to CLSI M100 ED34:2024 guidelines, except for CXI, GEN, AMC, and NAL, which were interpreted using EUCAST criteria. Resistance was defined by the following specific cut-off values: Nalidixic acid: MIC ≥32 µg/mL, Trimethoprim-Sulfamethoxazole: MIC ≥ 4/76 µg/mL (1:19 ratio) Amoxicillin-Clavulanic acid: MIC ≥32 µg/mL, Azithromycin: MIC ≥32 µg/ml.

2. In table 1, row 2 column 3, what is "{greater than or equal to}4/76"?

This value refers to the breakpoint for Trimethoprim-Sulfamethoxazole (Co-trimoxazole), where the ratio is 1:19. The breakpoint is ≥4/76 µg/mL. We have clarified this notation in the footnote of the new Table 1 to avoid confusion.

3. line 185 - 191, number of isolates should be shown together with rate, i.e. TET (90%, n=63). This applies to the entire manuscript.

We have revised the entire manuscript to consistently present the isolate count (n) next to every reported percentage. These changes have been applied extensively throughout the Abstract, the Results (both in the Antimicrobial Resistance Profiles and Genomic Features subsections), and the Discussion.

- A total of 72 isolates were recovered during a nationwide surveillance effort, among which (93%, n=67) were MDR based on antimicrobial susceptibility testing (disk diffusion and broth microdilution) results, including resistance to critically important agents such as quinolones, and highly important classes such as tetracyclines and sulfonamides.

- AST on the 72 S. Infantis isolates revealed significant resistance to commonly used antibiotics (Supplementary Table S1). Disk diffusion analysis Disk diffusion analysis (Figure 1) showed high resistance rates against TET (90.3%, n=65) and CIP (36.1%, n=26), with lower resistance observed for AMP (15.3%, n=11), CXI (11.1%, n=8), and CHL (8.3%, n=6). Minimal resistance was detected for GEN (5.5%, n=4) and CTR (1.4%, n=1), while no resistance was observed against MER. Additionally, broth microdilution results (Table 1) confirmed resistance to NAL (100%, n=72) and high resistance to TRS (95.8%, n=69), whereas. Conversely, all isolates were sensitive to AZI (Table 1).

- The IncFIB(pN55391) replicon, a key marker of pESI-like plasmids, was identified in 79% (n=15)8% of the isolates, emphasizing its role as a conserved genetic backbone. Additional plasmid types, such as Col(pHAD28) and IncI1-I(gamma), were found in 21%(n=4) and 11% (n=2) of the isolates, reflecting genetic variability.

- Antimicrobial susceptibility testing revealed that 93% of the studied Lebanese *S.* Infantis isolates were classified as MDR, with high resistance to TET (90%, n=65) and TRS (96%, n=69).

4. line 156 - 159, Table 2 and Figure 2: The authors defined that pESI-like plasmid is present in the isolate if "plasmid-specific markers, including piL, sogS, trbA, ipf, ipr2, ardA, and IncFIB (pN55391), with at least four of these markers needed to confirm the presence of the pESI-like plasmid". Using this definition, the authors therefore stated that all 19 isolates harbor pESI-like plasmid in Figure 2. Why the authors used this definition? Is it based on previous studies? Any reference? In this study, all isolates were sequenced by Illumina short read and genomes were assembled into draft genome level, which in nature limited its usage in detecting large pESI-like plasmids with a size of ~280kb. Therefore, a more stringent comparative sequence analysis and standard is required to determine if the isolates harbor pESI-like plasmid.

We agree that short-read (Illumina) sequencing cannot fully resolve the complete, closed structure of the ~280kb megaplasmid. However, our definition is based on the detection of a specific constellation of conserved pESI-diagnostic markers rather than the assembly of a single complete contig. This marker-based approach is a standard, validated method for detecting the pESI lineage in short-read data. To ensure our findings are robust and conservative, we have:

- **Updated the Methods (Bioinformatic analysis): We now explicitly justify our marker-based threshold and cite recent work by Li et al. (2024), who screened >500,000 Salmonella genomes for pESI using the same seven canonical markers (IncFIB(pN55391), *ardA, pilL, sogS, trbA, ipf, and ipr2*) and classified isolates as pESI-positive when ≥3 markers were present. In our study, we apply a more conservative operational criterion and consider isolates to harbor a pESI-like plasmid backbone when ≥4 of these canonical markers are detected with high identity and coverage. This choice is now clearly described as a pragmatic, conservative threshold for short-read assemblies rather than a universal formal definition.**
- **Refined the Terminology: We have adjusted the text throughout the manuscript to refer to the detection of a "pESI-like backbone" or "pESI-associated markers," accurately reflecting the genomic evidence provided by short-read assemblies.**
- **Comparative plasmid alignment (more stringent analysis): As an additional line of evidence, we performed a comparative genomic alignment (Figure 2A) of the largest plasmid contigs from 13 isolates against the reference pESI plasmid CP047882.1. This analysis shows that, despite assembly fragmentation, these contigs retain the conserved backbone structure and synteny characteristic of the international pESI**

lineage, supporting the marker-based inference of pESI-like plasmid backbones in our Lebanese *S*. Infantis isolates.

5. line 247 - 249: The conservativity of pESI marker should be determined by analysing all publically available complete pESI-like plasmid sequences, but not only by 19 isolates sequenced in this study.

We agree with the reviewer that establishing the conservation of diagnostic markers is essential for accurate plasmid identification. While an exhaustive analysis of all thousands of publicly available pESI-like sequences is beyond the scope of this study, we ensured the robustness of our marker selection by:

- **Utilizing Validated Markers: The diagnostic markers used in this study (*pilL, sogS, trbA, ipf, ipr2, ardA*, and IncFIB(pN55391)) were selected because they have been established as highly conserved "core" components of the pESI megaplasmid backbone in previous large-scale global genomic analyses of S. Infantis populations in Europe and the Americas (e.g. Alba et al., 2020; Li et al., 2024). These studies provide the primary evidence for their global conservation.**

- **Comparative Analysis with International References: In Figure 2A, we performed a comparative alignment of our isolates against representative, complete pESI-like plasmid sequences from diverse geographic regions, including Italy, the USA and Turkey confirming that our markers maintain high sequence identity and consistent synteny with international reference megaplasmids.**

6. Figure 3A: 13 S. Infantis isolates in this study were compared with international references. If all 19 isolates were determined to have pESI as shown in Figure 2, why only 13 were used for comparison? It will be more convincing if all the 19 isolates harbor a relative integral plasmid backbone as the 13 isolates shown in Figure 3A to conclude that all 19 isolates harbour pESI-like plasmid.

The selection of 13 isolates for the comparative genomic alignment in Figure 3A was based on a specific technical threshold for assembly contiguity to ensure a clear and interpretable representation of plasmid synteny.

As shown in our Supplementary Table S2, the assembly statistics for the 19 sequenced isolates exhibited a wide range of contiguity, with N50 values spanning from 106.9 kb to 694.3 kb and contig counts ranging from 29 to 109.

- **Technical Justification: For a comparative circular alignment (Circos plot), high fragmentation (low N50 and high contig counts) introduces significant visual gaps and "noise" that can lead to the false appearance of large deletions or structural**

rearrangements where none exist. To avoid this, we prioritized the 13 isolates with the highest assembly continuity (median N50 of 206.7 kb).

- Consistency of Data: While these 6 highly fragmented assemblies were excluded from the visual alignment for clarity, we verified that they are biologically consistent with the rest of the cohort. As detailed in Table 2, all 19 isolates regardless of their assembly N50, consistently harbored the conserved pESI-diagnostic marker constellation.

By applying this quality-based selection, we ensured that Figure 2A accurately represents the structural stability of the pESI-like backbone in our population without the artifacts inherent to fragmented short-read assemblies. We have updated the Figure 2 legend to explicitly state this selection criterion.

7. Figure 4: if all isolates had the same source of isolation, the source should not be shown in the figure to avoid redundancy.

In the revised version, we have removed the source-of-isolation information from the phylogenetic figure (previously Figure 4). All isolates in this dataset were indeed obtained from poultry sources, so displaying the source ring was redundant and did not add information. The updated figure now focuses on the core-genome relationships and AMR gene clusters only, and all corresponding references to the source ring have been removed from the figure and its legend.

8. line 301 - 303: How did the authors define the isolates have "clear phylogenetic relatedness" based on the cgMLST tree? There is no scale bar for the tree in Figure 4, nor did the authors show the snp distance of the isolates, included any reference genomes. The authors need to provide such information to make this statement.

We have revised our phylogenetic analysis to more clearly define the degree of relatedness among the Lebanese isolates. Specifically, we replaced the previous circular cgMLST plot with a rectangular phylogenetic tree (now Figure 3) in which branch lengths are proportional to core-genome allelic differences and a horizontal scale bar is provided. The figure legend now indicates that the red value "167" corresponds to the maximum core-genome allelic distance separating isolates within the main Lebanese cluster from more distantly related isolates. In the Results section "Phylogenetic Relationships and Antimicrobial Resistance Genes", we now state that most Lebanese isolates form a tight cluster with core-genome allelic distances ≤167, whereas distances to outlying isolates are substantially higher, consistent with clonal expansion of a dominant pESI-positive lineage. In addition, we have added a new Supplementary Figure S3 showing the pairwise core-genome SNP distance matrix underlying this tree. Together, these changes quantitatively define the "clear phylogenetic relatedness" among the poultry-associated isolates and address the reviewer's concerns.

9. Figure 5: This Figure mis-presented the information. The reviwer suggests the authors to present the following in the figure: the heatmap should consists of 1. presence of AMR gene in the isolate; and 2. if the isolate is resistant or sensitive to the antiboitic of the class. this is to discuss if WGS results tally with phenotypic results.

We agree that a direct comparison between genomic and phenotypic data is essential to validate the functional role of the identified resistance determinants. To address this, we have completely redesigned the heatmap (now Figure 4) to present a side-by-side analysis of the genotype and phenotype for each isolate. The updated figure now includes:

- **Genotype Columns: The presence or absence of specific AMR genes (e.g., *tet(A), sul1, blaTEM-1B*).**
- **Phenotype Columns: The corresponding antimicrobial susceptibility testing (AST) result (Resistant vs. Susceptible) for the representative antibiotic of each class.**

Furthermore, we have added a new paragraph to the Results where we explicitly discuss the concordance between the two datasets. Our analysis revealed a high degree of consistency, with 100% concordance observed for sulfonamides and quinolones, and 94.7% for tetracyclines. We have also updated the Discussion to emphasize that the identified pESI-like resistome is a reliable predictor of the MDR phenotypes observed in Lebanese poultry.

Re: Spectrum02418-25R2 (Molecular Characterization of pESI- like Megaplasmids in Salmonella Infantis from Poultry in Lebanon)

Dear Dr. Ghassan M Matar:

Thank you for the privilege of reviewing your work. Below you will find my comments, instructions from the Spectrum editorial office, and the reviewer comments.

Please return the manuscript within 60 days; if you cannot complete the modification within this time period, please contact me. If you do not wish to modify the manuscript and prefer to submit it to another journal, notify me immediately so that the manuscript may be formally withdrawn from consideration by Spectrum.

Revision Guidelines
To submit your modified manuscript, log into the submission site at https://spectrum.msubmit.net/cgi-bin/main.plex. Go to Author Tasks and click the appropriate manuscript title to begin. The information you entered when you first submitted the paper will be displayed; update this as necessary. Note the following requirements:

• Upload point-by-point responses to the issues raised by the reviewers in a file named "Response to Reviewers," NOT in your cover letter.
• Upload a compare copy of the manuscript (without figures) as a "Marked-Up Manuscript" file.
• Upload a clean .DOC/.DOCX version of the revised manuscript and remove the previous version.
• Each figure must be uploaded as a separate, editable, high-resolution file (TIFF or EPS preferred), and any multipanel figures must be assembled into one file.
• Any supplemental material intended for posting by ASM should be uploaded with their legends separate from the main manuscript. You can combine all supplemental material into one file (preferred) or split it into a maximum of 10 files with all associated legends included.

For complete guidelines on revision requirements, see our Submission and Review Process webpage. Submission of a paper that does not conform to guidelines may delay acceptance of your manuscript.

Data availability: ASM policy requires that data be available to the public upon online posting of the article, so please verify all links to sequence records, if present, and make sure that each number retrieves the full record of the data. If a new accession number is not linked or a link is broken, provide Spectrum production staff with the correct URL for the record. If the accession numbers for new data are not publicly accessible before the expected online posting of the article, publication may be delayed; please contact production staff (Spectrum@asmusa.org) immediately with the expected release date.

Publication Fees: For information on publication fees and which article types are subject to charges, visit our website. If your manuscript is accepted for publication and any fees apply, you will be contacted separately about payment during the production process; please follow the instructions in that e-mail. Arrangements for payment must be made before your article is published.

ASM Membership: Corresponding authors may join or renew ASM membership to obtain discounts on publication fees. Need to upgrade your membership level? Please contact Customer Service at Service@asmusa.org.

The ASM Journals program strives for constant improvement in our submission and publication process. Please tell us how we can improve your experience by taking this quick Author Survey.

Thank you for submitting your paper to Spectrum.

Sincerely,
John Osei Sekyere
Editor
Microbiology Spectrum

Reviewer #1 (Comments for the Author):

Molecular Characterization of pESI-like Megaplasmids in Salmonella Infantis from Poultry in Lebanon

This manuscript presents valuable genomic data on pESI-positive Salmonella Infantis from Lebanese poultry, filling an important regional gap in AMR surveillance. The study is generally well-designed and the findings are significant. However, several areas

require attention to strengthen the manuscript.

First, this reviewer identified the following potential issues or mistakes in the authors' responses to reviewer comments:
1. The % of TET resistance has listed 90.3% as well as 90%, and please just make them consistent.
2. bottom of page 2, what is ("79% (n=15)8%")? Please clarify.
3. The authors acknowledge that "an exhaustive analysis of all thousands of publicly available pESI-like sequences is beyond the scope of this study" but then claim their markers are validated based on previous studies. This is somewhat circular reasoning. The reviewer specifically asked them to validate marker conservation using public data, and the authors essentially declined while still claiming the markers are conserved.
4. The response refers to "Figure 3A" when discussing the comparative alignment, but earlier responses and the document structure suggest this should be "Figure 2A." Please make sure a correct reference to the figure is cited.
Regarding the revised manuscript, additional comments are provided below:
1. Study Design and Sampling Limitations: The single-month sampling period seems a significant limitation that deserves more prominent discussion earlier in the manuscript, not just in the limitations section. Consider adding this information to the Methods section introduction. In addition, the lack of farm-level metadata limits epidemiological interpretation. While you acknowledge this, consider discussing whether clonal clustering could reflect inter-farm transmission versus shared sources. Finally, the 8-year gap between sample collection (2017) and publication raises questions about current relevance. Address whether the AMR patterns observed remain representative of current conditions in Lebanon.
2. The rationale for selecting 19 of 72 isolates for WGS may need clarification. You state they were "randomly selected to represent genetic diversity based on phenotypic and genotypic characteristics," which seems contradictory. Please list some specific selection criteria that were used.

Thanks for addressing review comments made to the previous version. The manuscript has been improved, which can provide some useful information to the readers.

Reviewer #1 (Comments for the Author):

First, this reviewer identified the following potential issues or mistakes in the authors' responses to reviewer comments:

1. The % of TET resistance has listed 90.3% as well as 90%, and please just make them consistent. **We have standardized the report of tetracycline (TET) resistance to 90.3% (n=65) throughout the entire manuscript.**

2. bottom of page 2, what is ("79% (n=15)8%")? Please clarify. **This was a typographical error in the previous response document. In the revised manuscript, this value has been corrected to 89.5% (n=17) to accurately reflect the detection of the IncFIB(pN55391) replicon in 17 of the 19 sequenced isolates, as shown in Table 2 and the Results section.**

3. The authors acknowledge that "an exhaustive analysis of all thousands of publicly available pESI-like sequences is beyond the scope of this study" but then claim their markers are validated based on previous studies. This is somewhat circular reasoning. The reviewer specifically asked them to validate marker conservation using public data, and the authors essentially declined while still claiming the markers are conserved.

We appreciate the reviewer's clarification and agree that our previous wording did not sufficiently distinguish between *marker validation* and *marker application*. We did not intend to claim that marker conservation was newly validated within this study. Rather, we rely on diagnostic pESI markers that have already been established as conserved through prior large-scale public-data analyses, and we apply these markers as an operational definition to interrogate our Lebanese isolate collection.

Specifically, global conservation of the pESI backbone markers used here (*pilL, sogS, trbA, ipf, ipr2, ardA*, and IncFIB(pN55391)) has been demonstrated in previous studies that screened tens to hundreds of thousands of publicly available *S. Infantis* genomes (e.g., Alba et al., 2020; Li et al., 2024). These studies constitute the primary validation of marker conservation using public data, rather than the present work.

In our study, we therefore do not re-validate marker conservation globally, but instead apply these previously validated markers to identify pESI-like plasmid backbones in Lebanese isolates sequenced by short-read WGS. To support the appropriateness of this application, we additionally performed comparative alignments of representative Lebanese plasmid contigs against complete international pESI reference plasmids (Figure 2A), demonstrating conserved backbone synteny consistent with the established pESI lineage.

We have revised the manuscript to clarify this distinction and to avoid any implication that marker conservation was independently validated within this dataset.

4. The response refers to "Figure 3A" when discussing the comparative alignment, but earlier responses and the document structure suggest this should be "Figure 2A." Please make sure a correct reference to the figure is cited. **We have corrected the reference; the comparative genomic alignment is indeed Figure 2A. All figure citations in the manuscript have been cross-checked for accuracy.**

Regarding the revised manuscript, additional comments are provided below:

1. **Study Design and Sampling Limitations: The single-month sampling period seems a significant limitation that deserves more prominent discussion earlier in the manuscript, not just in the limitations section. Consider adding this information to the Methods section introduction. In addition, the lack of farm-level metadata limits epidemiological interpretation. While you acknowledge this, consider discussing whether clonal clustering could reflect inter-farm transmission versus shared sources. Finally, the 8-year gap between sample collection (2017) and publication raises questions about current relevance. Address whether the AMR patterns observed remain representative of current conditions in Lebanon.**

We have revised the manuscript to address these points as follows:

1- The single-month sampling period should be discussed earlier, not only in the limitations:

We have now clarified this point earlier in the manuscript by adding it to the materials and methods section. The revised text now states that all isolates were collected during a single sampling period in March 2017 and that the dataset therefore represents a cross-sectional snapshot of *Salmonella* Infantis circulating in Lebanese poultry farms at that time. This addresses the reviewer's request to make this limitation more prominent earlier in the manuscript:

# Materials and Methods (Section: Salmonella Infantis isolates): "A total of 72 *Salmonella enterica* serovar Infantis isolates were collected in March 2017 from poultry farms located in the Mount Lebanon region, as part of a nationwide agricultural surveillance campaign targeting zoonotic pathogens in livestock. All isolates were collected during a single sampling period (March 2017), providing a cross-sectional snapshot of *Salmonella* Infantis circulating in Lebanese poultry farms at that time."

2- Lack of farm-level metadata limits epidemiological interpretation:

We now address the lack of farm-level metadata in the Materials and Methods by clarifying that the high genetic relatedness observed among the isolates (≤167 allelic differences) could reflect inter-farm transmission, shared upstream sources such as hatcheries or feed

suppliers, or circulation within a broader regional poultry production network, rather than direct farm-to-farm spread alone.

# Materials and Methods: "As a result, downstream phylogenetic clustering could reflect either inter-farm transmission, shared upstream sources (e.g., hatcheries or feed suppliers), or circulation within a broader regional poultry production network rather than direct farm-to-farm spread."

# Results (Section: Phylogenetic Relationships and Antimicrobial Resistance Genes): "Within this main cluster, core-genome allelic distances between isolates were ≤167, whereas distances to more distantly related isolates outside the cluster were substantially higher, consistent with clonal expansion of a dominant pESI-positive lineage."

3- Scientific Justification of the 8-Year Gap:

To address the current relevance, we have added a dedicated analysis in the Discussion where we explicitly state that although the isolates were collected in 2017, the genomic and phenotypic patterns observed remain highly relevant to the current Lebanese and regional context. To support this statement, we cite recent studies from 2024–2025 in Lebanon, the United Arab Emirates, and Egypt showing continued circulation of pESI-positive MDR *S.* Infantis with similar resistance profiles. This supports the interpretation that the patterns observed in our 2017 dataset reflect persistent regional trends rather than isolated historical events.

# Discussion "Recent studies from 2024-2025 in the Middle East, including Lebanon, the UAE, and Egypt, continue to report a high prevalence of pESI-positive MDR S. Infantis with similar resistance profiles to those identified in our 2017 study (39–42). These consistent findings suggest that the pESI-like lineage is an ecologically stable and persistent threat in the region's poultry production chain. These consistent findings suggest that the pESI-like lineage is an ecologically stable and persistent threat in the region's poultry production chain".

# Discussion "Beyond Lebanon, recent Middle Eastern studies from Lebanon, Egypt, Iraq, and the Gulf region have reported a high prevalence of *S.* Infantis and MDR Salmonella along poultry value chains, with several genomic investigations from the United Arab Emirates and neighboring countries describing ST2283 *S.* Infantis carrying IncFIB(pN55391)-type pESI-like plasmids and extended-spectrum beta-lactamases such as blaCTX-M-65 (3,39–42). Taken together, these data indicate that the epidemiological pattern observed in Lebanese poultry, characterized by pESI-positive MDR S. Infantis with high fluoroquinolone and tetracycline resistance, is representative of a broader regional trend across the Eastern Mediterranean."

2. The rationale for selecting 19 of 72 isolates for WGS may need clarification. You state they were "randomly selected to represent genetic diversity based on phenotypic and genotypic

characteristics," which seems contradictory. Please list some specific selection criteria that were used. **The description of isolate selection has been revised in the Materials and Methods section to clarify that the 19 isolates were selected before sequencing primarily to capture the diversity of phenotypic antimicrobial susceptibility profiles observed in the 72-isolate collection. Selection considered variation in AST patterns, including the predominant MDR phenotype as well as less frequent resistance combinations involving quinolones, β-lactams, aminoglycosides, phenicols, and amoxicillin-clavulanic acid. The revised text now clarifies that the subset was selected to represent phenotypic diversity rather than through purely random sampling. Specifically, the selection was based on the representation of the following distinct AST patterns:**

- **Dominant Multidrug-Resistant (MDR) Profiles: Representation of the most prevalent resistance pattern characterized by resistance to tetracycline (TET), trimethoprim-sulfamethoxazole (TRS), and nalidixic acid (NAL). This includes isolates such as SAL_201703_01, SAL_201703_10, SAL_201703_11, SAL_201703_16, and SAL_201703_20.**
- **Extended Resistance Patterns: Selection of rare "extreme" phenotypes exhibiting additional resistance to critically or highly important antibiotics. This includes isolates such as SAL_201703_05 (resistant to ceftriaxone, cefoxitin, and ampicillin), SAL_201703_04 (resistant to gentamicin and ciprofloxacin), SAL_201703_07 (resistant to chloramphenicol), and SAL_201703_38 (resistant to ampicillin).**

# Abstract "WGS was performed on 19 isolates selected through a stratified approach to encompass all identified AMR phenotypes".

# Materials and Methods "Whole-genome sequencing (WGS) was performed on 19 MDR S. Infantis isolates selected from the collection of 72 to capture the diversity of antimicrobial susceptibility phenotypes observed. Selection criteria included representation of different AST patterns, including the predominant MDR phenotype (e.g., TET-TRS-NAL) as well as less frequent resistance combinations involving quinolones, β-lactams, aminoglycosides, phenicols, and amoxicillin-clavulanic acid (Table S1)."

Re: Spectrum02418-25R3 (Molecular Characterization of pESI- like Megaplasmids in Salmonella Infantis from Poultry in Lebanon)

Dear Dr. Ghassan M Matar:

Thank you for the privilege of reviewing your work. Below you will find my comments, instructions from the Spectrum editorial office, and the reviewer comments.

Please return the manuscript within 60 days; if you cannot complete the modification within this time period, please contact me. If you do not wish to modify the manuscript and prefer to submit it to another journal, notify me immediately so that the manuscript may be formally withdrawn from consideration by Spectrum.

Revision Guidelines
To submit your modified manuscript, log into the submission site at https://spectrum.msubmit.net/cgi-bin/main.plex. Go to Author Tasks and click the appropriate manuscript title to begin. The information you entered when you first submitted the paper will be displayed; update this as necessary. Note the following requirements:

• Upload point-by-point responses to the issues raised by the reviewers in a file named "Response to Reviewers," NOT in your cover letter.
• Upload a compare copy of the manuscript (without figures) as a "Marked-Up Manuscript" file.
• Upload a clean .DOC/.DOCX version of the revised manuscript and remove the previous version.
• Each figure must be uploaded as a separate, editable, high-resolution file (TIFF or EPS preferred), and any multipanel figures must be assembled into one file.
• Any supplemental material intended for posting by ASM should be uploaded with their legends separate from the main manuscript. You can combine all supplemental material into one file (preferred) or split it into a maximum of 10 files with all associated legends included.

For complete guidelines on revision requirements, see our Submission and Review Process webpage. Submission of a paper that does not conform to guidelines may delay acceptance of your manuscript.

Data availability: ASM policy requires that data be available to the public upon online posting of the article, so please verify all links to sequence records, if present, and make sure that each number retrieves the full record of the data. If a new accession number is not linked or a link is broken, provide Spectrum production staff with the correct URL for the record. If the accession numbers for new data are not publicly accessible before the expected online posting of the article, publication may be delayed; please contact production staff (Spectrum@asmusa.org) immediately with the expected release date.

Publication Fees: For information on publication fees and which article types are subject to charges, visit our website. If your manuscript is accepted for publication and any fees apply, you will be contacted separately about payment during the production process; please follow the instructions in that e-mail. Arrangements for payment must be made before your article is published.

ASM Membership: Corresponding authors may join or renew ASM membership to obtain discounts on publication fees. Need to upgrade your membership level? Please contact Customer Service at Service@asmusa.org.

The ASM Journals program strives for constant improvement in our submission and publication process. Please tell us how we can improve your experience by taking this quick Author Survey.

Thank you for submitting your paper to Spectrum.

Sincerely,
John Osei Sekyere
Editor
Microbiology Spectrum

Reviewer #1 (Comments for the Author):

Thanks the authors to address review comments made to the previous versions. No additional comments.

Reviewer #5 (Comments for the Author):

See attached

Barada et al. present characterization of *Salmonella* Infantis isolates carrying the pESI plasmid collected from poultry farms in Lebanon. The analysis is straightforward. Some of the claims are somewhat overstated and some important references are missing and should be included.

L63 – needs a ref (The rise…)

L67 – needs a ref (Beyond…)

L71 – add Aviv ref (ref #1) here and include Middle East in the list

McMillan papers from 2020 and 2022 should also be included in the introduction as they also describe rise of Infantis containing pESI. Further Cohen 2020 paper also characterizes Infantis and pESI in Israeli isolates, which may be geographically relevant to isolates found in Lebanon. For example, in L184, including Israeli isolates/plasmids in this context would be valuable.

L97 – how many farms? I understand that 'farm-level metadata was absent, but do the authors know how many farms? If so, needs to be included. Were these broiler-breeder farms or broiler farms? This is relevant to the statement L107-110 regarding downstream analysis.

L137. For clarity, reword to: Whole-genome sequencing (WGS) was performed on 19 MDR S. Infantis isolates. These were selected from the collection of 72 to capture the diversity of antimicrobial susceptibility phenotypes observed.

ST1 – suggest color-coding "S" and "R" cells so that the resistance patterns are easier to visualize for the reader.

L174 – cite figures in order they appear in text (in this case remove reference to the figure and say "genotype-phenotype concordance analysis was performed…" There are several figs references in the methods, which is odd.

L188 – is CP047882.1 a closed genome. Please state why it was selected as a reference

L225 – "co-occurred" is a vague term here. Were the AMR genes on the same contigs as the pESI markers?

Fig 2. I am not sure what I am looking at here. Please add size to A. In B, the size is 5100 Kb – is this the whole salmonella genome? Figure states it is the plasmid, which, according to most papers, is ~300 Kb. Would be most useful to show a higher resolution image of just the plasmid. Is this because all genomes are draft? The Cohen 2020 paper was a closed genome, so assume the plasmid was also closed – this might be a better reference sequence.

L298 – why were these regions selected? There have been plenty of pESI papers from other regions as well. Please provide a rational for selecting US, Italy, Turkey.

L305. Reword: were consistently present in isolates from all four countries…

L312 – I would be cautious here, without an outgroup, it may be difficult to say how closely clustered genomes are. Perhaps reword to say, "most isolates fell into a single cluster"

Fig 4 – it is hard to compare the genotypic and phenotypic results. Suggest putting a dot in the genotypic cells if the phenotype agreed.

L354: "Importantly, the role of pESI-like megaplasmid as a driver of both antimicrobial resistance and virulence was clearly demonstrated, consistent with global reports and emphasizing its critical role in Lebanese poultry production systems" I don't believe this has been addressed in the paper. They didn't look at virulence. "A driver of" is vague given that the authors didn't link AMR genes with pESI itself.

L364: "These patterns reflect the extensive use of these antibiotics in Lebanese agriculture and align with global reports identifying poultry production systems as key reservoirs for MDR pathogens (3,25)." Please provide refs for TET and and TRS use in Lebanese poultry production. Further "global" has only referred to papers from Lebanon and Hungary (also referred to as global context on L405). Generally, globally, antibiotic stewardship has improved. An alternative hypothesis to AMR is that Infantis (due to pESI or otherwise) is better adapted to chickens and/or chicken farm environments (i.e. authors comments in L374-378)

L371 – ref 30 is 24 years old, is this still relevant to current fluoroquinolone resistance incidence?

The discussion is quite long, please consider shortening

WGS data should be uploaded to a repository – eg. NCBI or Enterobase

Thanks for addressing review comments made to the previous version. The manuscript has been improved, which can provide some useful information to the readers.

Barada et al. present characterization of Salmonella Infantis isolates carrying the pESI plasmid collected from poultry farms in Lebanon. The analysis is straightforward. Some of the claims are somewhat overstated and some important references are missing and should be included.

L63 – needs a ref (The rise…)/L67 – needs a ref (Beyond…)

We have added appropriate references to support both statements. Specifically, the sentence describing the rise of MDR *S.* Infantis in association with pESI/pESI-like megaplasmids is now supported by studies documenting the role and dissemination of pESI-like plasmids in poultry-associated *S.* Infantis lineages. We also added references to support the statement that pESI-like plasmids may encode adaptive traits beyond antimicrobial resistance, including tolerance to heavy metals and disinfectant-associated stressors, which may contribute to bacterial persistence in poultry-production environments.

L71 – add Aviv ref (ref #1) here and include Middle East in the list.

We have revised the sentence to include the Middle East among the regions where pESI-positive *S.* Infantis has been reported and added the Aviv et al. reference, as suggested.

McMillan papers from 2020 and 2022 should also be included in the introduction as they also describe rise of Infantis containing pESI. Further Cohen 2020 paper also characterizes Infantis and pESI in Israeli isolates, which may be geographically relevant to isolates found in Lebanon. For example, in L184, including Israeli isolates/plasmids in this context would be valuable.

We have revised the Introduction to include the McMillan et al. 2020 and 2022 studies, which describe the emergence and increasing prevalence of pESI-positive S. Infantis in North American poultry-associated sources. We also added additional regional context by citing studies describing pESI-positive *Salmonella* from the Eastern Mediterranean, including the Cohen et al. study, to emphasize the relevance of geographically proximate pESI-positive lineages to Lebanon. In addition, we revised the Comparative Plasmid Alignment section to clarify the rationale for the reference sequences used in our analysis. Specifically, we now state that reference sequences were selected to represent well-characterized pESI/pESI-like plasmids from geographically and epidemiologically relevant poultry-associated lineages, while prioritizing publicly available complete or high-quality assemblies suitable for comparative alignment. Accordingly, we retained the representative sequences from Turkey, Italy, and the United States because they provided suitable complete or high-quality pESI/pESI-like datasets for direct visualization and comparison with the Lebanese assemblies. This rationale has been clarified in the revised manuscript.

L97 – how many farms? I understand that 'farm-level metadata was absent, but do the authors know how many farms? If so, needs to be included. Were these broiler-breeder farms or broiler farms? This is relevant to the statement L107-110 regarding downstream analysis.

We clarified in the revised Materials and Methods that the isolates were collected from multiple poultry farms in the Mount Lebanon region; however, detailed farm-level metadata were not available for the present analysis. Specifically, the exact number of farms, individual farm identifiers, and production type, including whether the farms were broiler or broiler-breeder farms, could not be verified from the available dataset.

To avoid over interpretation, we revised the manuscript to state that the isolates were analyzed collectively at the regional level. We also modified the downstream phylogenetic interpretation in the Results and Discussion to clarify that clustering could not be attributed to specific farms, production types, or direct farm-to-farm transmission. Instead, relatedness among isolates is now interpreted more cautiously as reflecting circulation of a closely related pESI-positive lineage within the sampled regional poultry population, with possible contribution from shared upstream sources such as hatcheries or feed suppliers.

L137. For clarity, reword to: Whole-genome sequencing (WGS) was performed on 19 MDR S. Infantis isolates. These were selected from the collection of 72 to capture the diversity of antimicrobial susceptibility phenotypes observed.

We have revised the sentence in the Whole-Genome Sequencing subsection for clarity, as recommended. The revised sentence now reads: "Whole-genome sequencing (WGS) was performed on 19 MDR *S.* Infantis isolates. These were selected from the collection of 72 to capture the diversity of antimicrobial susceptibility phenotypes observed."

ST1 – suggest color-coding "S" and "R" cells so that the resistance patterns are easier to visualize for the reader.

Supplementary Table S1 has been revised to color-code the antimicrobial susceptibility results. Specifically, resistant (R) and susceptible (S) cells in the antimicrobial columns were highlighted using distinct colors to improve visualization of resistance patterns across isolates. The Supplementary Table S1 legend was also updated to clarify the color-coding scheme.

L174 – cite figures in order they appear in text (in this case remove reference to the figure and say "genotype-phenotype concordance analysis was performed…" There are several figs references in the methods, which is odd.

We have removed figure citations from the Materials and Methods section to ensure that figures are cited in the order in which they appear in the Results. Specifically, the reference to Figure 4 in the genotype–phenotype concordance methods was removed, and the sentence was revised to state that genotype–phenotype concordance analysis was performed by

integrating AMR gene/mutation presence with the corresponding phenotypic susceptibility profiles. We also removed other figure citations from the Methods section where appropriate, so that figure references now appear sequentially in the Results.

L188 – is CP047882.1 a closed genome. Please state why it was selected as a reference.

We have clarified in the Comparative Plasmid Alignment section that CP047882.1 corresponds to the complete pESI plasmid sequence from *S.* Infantis strain 119944. We selected this sequence as the reference because it is a publicly available, well-annotated complete pESI plasmid deposited in NCBI and has been used in previous studies for pESI-like plasmid screening and comparative analyses.

L225 – "co-occurred" is a vague term here. Were the AMR genes on the same contigs as the pESI markers?

We have revised the manuscript to more accurately describe the genomic relationship between the AMR determinants and the pESI-like backbone markers.

We would like to clarify our findings with the following technical evidence from our assembly analysis:

- **Assembly Fragmentation and Linkage: Our isolates were sequenced using Illumina short-read technology, which resulted in varying levels of assembly fragmentation across the 19 isolates. As detailed in Supplementary Table S2, assembly contiguity ranged from highly contiguous (e.g., isolate SAMN24966743 with 29 contigs and an N50 of 694,304 bp) to more fragmented (e.g., isolate SAMN24966749 with 109 contigs and an N50 of 123,330 bp). Because pESI-like megaplasmids are large, approximately 285 kb, short-read assemblies may split the plasmid backbone across multiple contigs, preventing full circular reconstruction from short reads alone.**

- **Inference from Reference Mapping: Because the assemblies were fragmented, plasmid-level localization could not be determined solely from single-contig reconstruction. Therefore, we used reference-guided alignment to further assess the relationship between AMR determinants and the pESI-like backbone (Figure 2). Contigs carrying diagnostic pESI markers, as well as contigs carrying accessory AMR genes including sul1, tet(A), and aadA, mapped with high nucleotide identity and coverage to corresponding regions of the complete pESI reference sequence CP047882.1. This supports an association between these AMR determinants and the pESI-like reference backbone.**

- **Revised Terminology: To maintain scientific rigor, we replaced "co-occurred" with more precise wording, including "detected among isolates carrying pESI-like**

markers" and, where supported by reference-guided mapping, "inferred plasmid-borne association." This wording reflects the observed association while acknowledging that full physical circularization was limited by short-read data.

- Updated Limitations: We have explicitly added this point to the "Limitations" section, noting that definitive physical linkage for all isolates would require long-read sequencing (e.g., Oxford Nanopore).

Fig 2. I am not sure what I am looking at here. Please add size to A. In B, the size is 5100 Kb – is this the whole salmonella genome? Figure states it is the plasmid, which, according to most papers, is ~300 Kb. Would be most useful to show a higher resolution image of just the plasmid. Is this because all genomes are draft? The Cohen 2020 paper was a closed genome, so assume the plasmid was also closed – this might be a better reference sequence.

We clarified the Figure 2 legend to better distinguish between the two panels. The scale is already shown in both Figure 2A and Figure 2B; however, we agree that the previous legend did not make the difference between the panels sufficiently clear.

Figure 2A: represents the alignment of Lebanese and international pESI-like sequences against the complete pESI plasmid reference CP047882.1 from S. Infantis strain 119944. We have now added the approximate size of this reference plasmid to the legend, indicating that CP047882.1 is approximately 285 kb in length. Because the Lebanese isolates were generated using Illumina short-read sequencing, the Lebanese sequences shown in Panel A represent pESI-like regions/contigs aligned to the complete reference plasmid rather than fully closed Lebanese plasmids.

Figure 2B: in contrast, represents a whole-genome AMR gene content comparison, not a plasmid-size map. Therefore, the ~5.1 Mb scale corresponds to the full *Salmonella* genome/assembly context and should not be interpreted as the size of the pESI plasmid. We revised the Figure 2 title and legend to clarify that Panel A shows the pESI plasmid-based alignment, whereas Panel B shows genome-level AMR gene content across selected Lebanese and international isolates. We also improved the resolution and quality of Figure 2 to enhance readability of the scale, labels, and annotated genomic features.

L298 – why were these regions selected? There have been plenty of pESI papers from other regions as well. Please provide a rational for selecting US, Italy, Turkey.

We have clarified the rationale for selecting Turkey, Italy, and the United States as comparator regions in the revised manuscript. These regions were not intended to represent the full global diversity of pESI-positive S. Infantis, but were selected as representative datasets based on both geographic/epidemiological relevance and sequence suitability for comparative plasmid alignment. Italy and Turkey were included to provide Mediterranean and geographically proximate regional context relevant to Lebanon, while the United States

was included because pESI-positive poultry-associated S. Infantis lineages have been well characterized there. In addition, the selected datasets included publicly available complete or high-quality pESI/pESI-like sequences suitable for direct comparative visualization using mummer2circos/Circos. We have revised the manuscript to clarify this rationale.

L305. Reword: were consistently present in isolates from all four countries…

We have revised the sentence for clarity as recommended. The revised sentence now reads: "While *tet(A), floR, and ANT(3'')-IIa*, among others, were consistently present in isolates from all four countries, other genes such as *blaTEM-1* and *aadA* were geographically restricted."

L312 – I would be cautious here, without an outgroup, it may be difficult to say how closely clustered genomes are. Perhaps reword to say, "most isolates fell into a single cluster"

We have revised the Results section to avoid over interpreting the degree of relatedness and now state that "most isolates fell into a single cluster within the sampled Lebanese poultry associated population." We also softened the downstream interpretation to describe the presence of a dominant cluster within the sampled regional poultry population rather than making stronger claims about close clustering or clonal expansion.

Fig 4 – it is hard to compare the genotypic and phenotypic results. Suggest putting a dot in the genotypic cells if the phenotype agreed.

We revised Figure 4 to improve the comparison between genotypic and phenotypic antimicrobial resistance results. Specifically, we added white dots within genotypic cells where the detected AMR determinant agreed with the corresponding resistant phenotype. This allows genotype–phenotype concordance to be identified more easily across antimicrobial classes. The Figure 4 legend was also revised to clarify that dotted genotypic cells indicate concordance between the presence of a resistance determinant and resistance to the corresponding antimicrobial agent.

L354: "Importantly, the role of pESI-like megaplasmid as a driver of both antimicrobial resistance and virulence was clearly demonstrated, consistent with global reports and emphasizing its critical role in Lebanese poultry production systems" I don't believe this has been addressed in the paper. They didn't look at virulence. "A driver of" is vague given that the authors didn't link AMR genes with pESI itself.

We agree that the original wording was too strong. Virulence was not experimentally assessed in this study, and because our genomes were generated using short-read sequencing, we could not definitively resolve the physical linkage between all AMR genes and the pESI-like backbone. Therefore, we revised the Discussion to remove the statement that pESI was "a driver" of both antimicrobial resistance and virulence. The revised text now states more cautiously that the widespread detection of pESI-like markers together with AMR

determinants supports an association between this megaplasmid lineage and antimicrobial resistance in Lebanese poultry.

L364: "These patterns reflect the extensive use of these antibiotics in Lebanese agriculture and align with global reports identifying poultry production systems as key reservoirs for MDR pathogens (3,25)." Please provide refs for TET and and TRS use in Lebanese poultry production. Further "global" has only referred to papers from Lebanon and Hungary (also referred to as global context on L405). Generally, globally, antibiotic stewardship has improved. An alternative hypothesis to AMR is that Infantis (due to pESI or otherwise) is better adapted to chickens and/or chicken farm environments (i.e. authors comments in L374-378)

We agree that the original wording was too strong because antimicrobial usage data were not available for the sampled farms. Therefore, we revised the Discussion to avoid directly attributing the observed tetracycline and trimethoprim-sulfamethoxazole resistance patterns to specific antimicrobial use practices in Lebanese poultry production.

The revised text now states that these resistance patterns are consistent with previous reports of high tetracycline and trimethoprim-sulfamethoxazole resistance among poultry-associated bacteria in Lebanon and may reflect selective pressures within poultry production systems. We also added a cautionary statement clarifying that, in the absence of farm-level antimicrobial usage data, these patterns cannot be directly attributed to specific antimicrobial use practices.

In response to the reviewer's suggestion, we further added the alternative, non-mutually exclusive explanation that *S*. Infantis, potentially through pESI-like plasmid carriage or other adaptive traits, may be well adapted to chickens and poultry-production environments. We also revised the references supporting this statement to include Lebanon-relevant poultry resistance/residue studies rather than relying only on broader global references.

L371 – ref 30 is 24 years old, is this still relevant to current fluoroquinolone resistance incidence?

We agree that the 2002 reference was outdated for supporting current fluoroquinolone resistance incidence. Therefore, we removed this reference from the statement discussing nalidixic acid/quinolone resistance. The revised sentence is now supported by more recent and directly relevant studies already cited in the manuscript, including recent reports and reviews describing quinolone resistance and MDR patterns among poultry-associated *S*. Infantis from the Middle East, Europe, and the United States.

The discussion is quite long, please consider shortening

We have shortened and streamlined the Discussion by merging overlapping paragraphs, reducing repetitive statements, and improving the flow of the interpretation.

WGS data should be uploaded to a repository – eg. NCBI or Enterobase

The WGS data have already been deposited in the NCBI Sequence Read Archive. We have included a Data Availability section in the revised manuscript specifying the BioProject accession number and the associated sequencing run accessions for the 19 Lebanese S. Infantis isolates analyzed in this study. The raw Illumina sequencing reads are available under BioProject PRJNA293224, with the corresponding SRR accessions listed in the manuscript.

Re: Spectrum02418-25R4 (Molecular Characterization of pESI- like Megaplasmids in Salmonella Infantis from Poultry in Lebanon)

Dear Dr. Ghassan M Matar:

Your manuscript has been accepted, and I am forwarding it to the ASM production staff for publication. Your paper will first be checked to make sure all elements meet the technical requirements. ASM staff will contact you if anything needs to be revised before copyediting and production can begin. Otherwise, you will be notified when your proofs are ready to be viewed.

Data Availability: ASM policy requires that data be available to the public upon online posting of the article, so please verify all links to sequence records, if present, and make sure that each number retrieves the full record of the data. If a new accession number is not linked or a link is broken, provide production staff with the correct URL for the record. If the accession numbers for new data are not publicly accessible before the expected online posting of the article, publication may be delayed; please contact ASM production staff immediately with the expected release date.

Publication Fees: For information on publication fees and which article types have charges, please visit our website. We have partnered with Copyright Clearance Center (CCC) to collect author charges. If fees apply to your paper, you will receive a message from no-reply@copyright.com with further instructions. For questions related to paying charges through RightsLink, please contact CCC at ASM_Support@copyright.com or toll free at +1-877-622-5543. CCC makes every attempt to respond to all emails within 24 hours.

ASM Membership: Corresponding authors may join or renew ASM membership to obtain discounts on publication fees. Need to upgrade your membership level? Please contact Customer Service at Service@asmusa.org.

Embargo Policy: A press release may be issued as soon as the manuscript is posted on the Spectrum Latest Articles webpage. The corresponding author will receive an email with the subject line "ASM Journals Author Services Notification" when the article is available online.

The ASM Journals program strives for constant improvement in our submission and publication process. Please tell us how we can improve your experience by taking this quick Author Survey.

Thank you for submitting your paper to Spectrum.

Sincerely,
John Osei Sekyere
Editor
Microbiology Spectrum

Reviewer #5 (Comments for the Author):

Thank you to the authors for responding to my previous comments; the manuscript is much improved.
