# Supplementary material for: Molecular characterization of pESI-like megaplasmids in Salmonella Infantis from poultry in Lebanon
Source: Microbiol Spectr. 2026 Jul 6;14(8):e02418-25. doi: 10.1128/spectrum.02418-25 (PMC13435791; doi:10.1128/spectrum.02418-25)
Supplement: Supplementary file 2 [file spectrum.02418-25-s0001.docx]

**Supplementary Table S1**

**Table S1.** Antimicrobial Susceptibility Profiles of S. Infantis Isolates Based on Disk Diffusion and Broth Microdilution Methods. Internal ID: Unique identifier for each Salmonella isolate. CTR (Ceftriaxone), CXI (Cefoxitin), MER (Meropenem), AMP (Ampicillin), CHL (Chloramphenicol), GEN (Gentamicin), CIP (Ciprofloxacin), TET (Tetracycline), TRS (Trimethoprim-Sulfamethoxazole), AMC (Amoxicillin-Clavulanic Acid), AZI (Azithromycin), NAL (Nalidixic Acid), MDR (Multidrug-Resistant). Cells were color-coded to improve visualization of susceptibility patterns, with resistant (R) phenotypes highlighted in yellow and susceptible (S) phenotypes highlighted in red.

| **Internal ID** | **Source** | **SEQ** | **SAMN** | **SRR Run** | **CTR** | **CXI** | **MER** | **AMP** | **CHL** | **GEN** | **CIP** | **TET** | **TRS** | **AMC** | **AZI** | **NAL** |
| --- | --- | --- | --- | --- | --- | --- | --- | --- | --- | --- | --- | --- | --- | --- | --- | --- |
| **SAL_201703_01** | **Poultry** | **Yes** | **SAMN24966732** | **SRR17614876** | **S** | **S** | **S** | **S** | **S** | **S** | **S** | **R** | **R** | **S** | **S** | **R** |
| SAL_201703_02 | **Poultry** | No | . | . | **S** | **S** | **S** | **S** | **R** | **S** | **S** | **R** | **R** | **S** | **S** | **R** |
| **SAL_201703_03** | **Poultry** | **Yes** | **SAMN24966733** | **SRR17614877** | **S** | **S** | **S** | **S** | **S** | **R** | **R** | **S** | **R** | **S** | **S** | **R** |
| **SAL_201703_04** | **Poultry** | **Yes** | **SAMN24966734** | **SRR17614907** | **S** | **R** | **S** | **R** | **S** | **R** | **R** | **R** | **R** | **R** | **S** | **R** |
| **SAL_201703_05** | **Poultry** | **Yes** | **SAMN24966735** | **SRR17614906** | **R** | **R** | **S** | **R** | **S** | **R** | **R** | **R** | **R** | **R** | **S** | **R** |
| SAL_201703_06 | **Poultry** | No | . | . | **S** | **S** | **S** | **S** | **S** | **S** | **S** | **R** | **R** | **S** | **S** | **R** |
| **SAL_201703_07** | **Poultry** | **Yes** | **SAMN24966736** | **SRR17614878** | **S** | **S** | **S** | **R** | **R** | **S** | **S** | **R** | **R** | **S** | **S** | **R** |
| SAL_201703_08 | **Poultry** | No | . | . | **S** | **S** | **S** | **S** | **S** | **S** | **S** | **R** | **R** | **S** | **S** | **R** |
| SAL_201703_09 | **Poultry** | No | . | . | **S** | **S** | **S** | **S** | **S** | **S** | **S** | **S** | **R** | **S** | **S** | **R** |
| **SAL_201703_10** | **Poultry** | **Yes** | **SAMN24966737** | **SRR17614879** | **S** | **S** | **S** | **S** | **S** | **S** | **S** | **R** | **R** | **S** | **S** | **R** |
| **SAL_201703_11** | **Poultry** | **Yes** | **SAMN11310165** | **SRR8833924** | **S** | **S** | **S** | **S** | **S** | **S** | **S** | **R** | **R** | **S** | **S** | **R** |
| SAL_201703_12 | **Poultry** | No | . | . | **S** | **S** | **S** | **R** | **R** | **S** | **R** | **R** | **R** | **S** | **S** | **R** |
| SAL_201703_13 | **Poultry** | No | . | . | **S** | **R** | **S** | **S** | **S** | **S** | **R** | **R** | **R** | **S** | **S** | **R** |
| SAL_201703_14 | **Poultry** | No | . | . | **S** | **S** | **S** | **S** | **S** | **S** | **R** | **R** | **R** | **S** | **S** | **R** |
| SAL_201703_15 | **Poultry** | No | . | . | **S** | **S** | **S** | **S** | **S** | **S** | **S** | **R** | **R** | **S** | **S** | **R** |
| **SAL_201703_16** | **Poultry** | **Yes** | **SAMN11310164** | **SRR8833925** | **S** | **S** | **S** | **S** | **S** | **S** | **S** | **R** | **R** | **S** | **S** | **R** |
| SAL_201703_17 | **Poultry** | No | . | . | **S** | **S** | **S** | **S** | **S** | **S** | **S** | **R** | **R** | **S** | **S** | **R** |
| SAL_201703_18 | **Poultry** | No | . | . | **S** | **S** | **S** | **S** | **S** | **S** | **S** | **R** | **R** | **S** | **S** | **R** |
| SAL_201703_19 | **Poultry** | No | . | . | **S** | **S** | **S** | **S** | **S** | **S** | **S** | **R** | **R** | **S** | **S** | **R** |
| **SAL_201703_20** | **Poultry** | **Yes** | **SAMN24966739** | **SRR17614905** | **S** | **S** | **S** | **S** | **S** | **S** | **S** | **R** | **R** | **S** | **S** | **R** |
| SAL_201703_21 | **Poultry** | No | . | . | **S** | **S** | **S** | **S** | **S** | **S** | **S** | **R** | **R** | **S** | **S** | **R** |
| SAL_201703_22 | **Poultry** | No | . | . | **S** | **S** | **S** | **S** | **S** | **S** | **R** | **R** | **R** | **S** | **S** | **R** |
| **SAL_201703_23** | **Poultry** | **Yes** | **SAMN24966740** | **SRR17614903** | **S** | **S** | **S** | **S** | **S** | **S** | **S** | **R** | **R** | **S** | **S** | **R** |
| **SAL_201703_24** | **Poultry** | **Yes** | **SAMN24966741** | **SRR17614881** | **S** | **S** | **S** | **S** | **S** | **S** | **R** | **R** | **R** | **S** | **S** | **R** |
| **SAL_201703_25** | **Poultry** | **Yes** | **SAMN24966742** | **SRR17614882** | **S** | **S** | **S** | **S** | **S** | **S** | **S** | **R** | **R** | **S** | **S** | **R** |
| **SAL_201703_26** | **Poultry** | **Yes** | **SAMN24966743** | **SRR17614883** | **S** | **S** | **S** | **S** | **S** | **S** | **S** | **R** | **R** | **S** | **S** | **R** |
| SAL_201703_27 | **Poultry** | No | . | . | **S** | **R** | **S** | **S** | **S** | **S** | **S** | **R** | **R** | **S** | **S** | **R** |
| SAL_201703_28 | **Poultry** | No | . | . | **S** | **S** | **S** | **S** | **S** | **S** | **R** | **R** | **R** | **S** | **S** | **R** |
| SAL_201703_29 | **Poultry** | No | . | . | **S** | **S** | **S** | **S** | **S** | **S** | **S** | **R** | **R** | **S** | **S** | **R** |
| SAL_201703_30 | **Poultry** | No | . | . | **S** | **S** | **S** | **S** | **S** | **S** | **S** | **R** | **R** | **S** | **S** | **R** |
| SAL_201703_31 | **Poultry** | No | . | . | **S** | **R** | **S** | **S** | **S** | **S** | **R** | **R** | **R** | **S** | **S** | **R** |
| **SAL_201703_32** | **Poultry** | **Yes** | **SAMN24966744** | **SRR17614902** | **S** | **S** | **S** | **S** | **S** | **S** | **S** | **R** | **R** | **S** | **S** | **R** |
| SAL_201703_33 | **Poultry** | No | . | . | **S** | **S** | **S** | **S** | **S** | **S** | **S** | **R** | **R** | **S** | **S** | **R** |
| SAL_201703_34 | **Poultry** | No | . | . | **S** | **S** | **S** | **S** | **S** | **S** | **R** | **R** | **R** | **S** | **S** | **R** |
| SAL_201703_35 | **Poultry** | No | . | . | **S** | **S** | **S** | **S** | **S** | **S** | **S** | **R** | **R** | **S** | **S** | **R** |
| **SAL_201703_36** | **Poultry** | **Yes** | **SAMN24966745** | **SRR17614901** | **S** | **S** | **S** | **S** | **S** | **S** | **S** | **R** | **R** | **S** | **S** | **R** |
| SAL_201703_37 | **Poultry** | No | . | . | **S** | **R** | **S** | **S** | **S** | **S** | **R** | **R** | **R** | **S** | **S** | **R** |
| **SAL_201703_38** | **Poultry** | **Yes** | **SAMN24966746** | **SRR17614884** | **S** | **S** | **S** | **S** | **S** | **S** | **R** | **R** | **R** | **S** | **S** | **R** |
| SAL_201703_39 | **Poultry** | No | . | . | **S** | **R** | **S** | **S** | **R** | **S** | **R** | **R** | **R** | **S** | **S** | **R** |
| **SAL_201703_40** | **Poultry** | **Yes** | **SAMN24966747** | **SRR17614885** | **S** | **S** | **S** | **S** | **S** | **S** | **S** | **S** | **R** | **R** | **S** | **R** |
| **SAL_201703_41** | **Poultry** | **Yes** | **SAMN24966748** | **SRR17614886** | **S** | **S** | **S** | **S** | **S** | **S** | **S** | **R** | **R** | **S** | **S** | **R** |
| SAL_201703_42 | **Poultry** | No | . | . | **S** | **S** | **S** | **R** | **S** | **S** | **S** | **R** | **R** | **S** | **S** | **R** |
| SAL_201703_43 | **Poultry** | No | . | . | **S** | **S** | **S** | **S** | **S** | **S** | **R** | **R** | **R** | **S** | **S** | **R** |
| SAL_201703_44 | **Poultry** | No | . | . | **S** | **S** | **S** | **R** | **S** | **S** | **R** | **R** | **R** | **R** | **S** | **R** |
| SAL_201703_45 | **Poultry** | No | . | . | **S** | **S** | **S** | **R** | **S** | **S** | **R** | **R** | **R** | **S** | **S** | **R** |
| SAL_201703_46 | **Poultry** | No | . | . | **S** | **S** | **S** | **S** | **S** | **S** | **S** | **R** | **R** | **S** | **S** | **R** |
| **SAL_201703_47** | **Poultry** | **Yes** | **SAMN24966749** | **SRR17614900** | **S** | **S** | **S** | **S** | **S** | **S** | **S** | **R** | **R** | **S** | **S** | **R** |
| SAL_201703_48 | **Poultry** | No | . | . | **S** | **S** | **S** | **S** | **S** | **S** | **R** | **R** | **R** | **S** | **S** | **R** |
| SAL_201703_49 | **Poultry** | No | . | . | **S** | **S** | **S** | **S** | **S** | **S** | **S** | **R** | **R** | **S** | **S** | **R** |
| SAL_201703_50 | **Poultry** | No | . | . | **S** | **S** | **S** | **R** | **R** | **S** | **R** | **R** | **R** | **S** | **S** | **R** |
| SAL_201703_51 | **Poultry** | No | . | . | **S** | **S** | **S** | **S** | **S** | **S** | **S** | **R** | **R** | **S** | **S** | **R** |
| SAL_201703_52 | **Poultry** | No | . | . | **S** | **S** | **S** | **R** | **S** | **S** | **R** | **R** | **R** | **S** | **S** | **R** |
| SAL_201703_53 | **Poultry** | No | . | . | **S** | **S** | **S** | **S** | **S** | **S** | **S** | **R** | **S** | **S** | **S** | **R** |
| SAL_201703_54 | **Poultry** | No | . | . | **S** | **R** | **S** | **S** | **S** | **S** | **R** | **R** | **R** | **S** | **S** | **R** |
| SAL_201703_55 | **Poultry** | No | . | . | **S** | **S** | **S** | **S** | **S** | **S** | **S** | **S** | **S** | **S** | **S** | **R** |
| SAL_201703_56 | **Poultry** | No | . | . | **S** | **S** | **S** | **S** | **S** | **S** | **S** | **R** | **S** | **S** | **S** | **R** |
| SAL_201703_57 | **Poultry** | No | . | . | **S** | **S** | **S** | **S** | **S** | **S** | **R** | **R** | **R** | **S** | **S** | **R** |
| SAL_201703_58 | **Poultry** | No | . | . | **S** | **S** | **S** | **S** | **S** | **S** | **R** | **R** | **R** | **S** | **S** | **R** |
| SAL_201703_59 | **Poultry** | No | . | . | **S** | **S** | **S** | **S** | **S** | **S** | **S** | **R** | **R** | **S** | **S** | **R** |
| SAL_201703_60 | **Poultry** | No | . | . | **S** | **S** | **S** | **S** | **S** | **S** | **R** | **R** | **R** | **S** | **S** | **R** |
| SAL_201703_61 | **Poultry** | No | . | . | **S** | **S** | **S** | **S** | **S** | **S** | **S** | **R** | **R** | **S** | **S** | **R** |
| SAL_201703_62 | **Poultry** | No | . | . | **S** | **S** | **S** | **R** | **S** | **S** | **S** | **S** | **R** | **S** | **S** | **R** |
| SAL_201703_63 | **Poultry** | No | . | . | **S** | **S** | **S** | **S** | **S** | **S** | **S** | **R** | **R** | **S** | **S** | **R** |
| SAL_201703_64 | **Poultry** | No | . | . | **S** | **S** | **S** | **S** | **S** | **S** | **R** | **S** | **R** | **S** | **S** | **R** |
| SAL_201703_65 | **Poultry** | No | . | . | **S** | **S** | **S** | **S** | **S** | **R** | **S** | **R** | **R** | **S** | **S** | **R** |
| SAL_201703_66 | **Poultry** | No | . | . | **S** | **S** | **S** | **S** | **S** | **S** | **S** | **R** | **R** | **S** | **S** | **R** |
| SAL_201703_67 | **Poultry** | No | . | . | **S** | **S** | **S** | **S** | **S** | **S** | **S** | **R** | **R** | **S** | **S** | **R** |
| SAL_201703_68 | **Poultry** | No | . | . | **S** | **S** | **S** | **R** | **R** | **S** | **S** | **R** | **R** | **S** | **S** | **R** |
| SAL_201703_69 | **Poultry** | No | . | . | **S** | **S** | **S** | **S** | **S** | **S** | **S** | **R** | **R** | **S** | **S** | **R** |
| SAL_201703_70 | **Poultry** | No | . | . | **S** | **S** | **S** | **S** | **S** | **S** | **S** | **R** | **R** | **S** | **S** | **R** |
| SAL_201703_71 | **Poultry** | No | . | . | **S** | **S** | **S** | **S** | **S** | **S** | **S** | **R** | **R** | **R** | **S** | **R** |
| SAL_201703_72 | **Poultry** | No | . | . | **S** | **S** | **S** | **S** | **S** | **S** | **R** | **S** | **R** | **S** | **S** | **R** |
| Incidence (%R) | **-** | - | - | - | **1.40%** | **11.11%** | **0%** | **15.30%** | **8.30%** | **5.50%** | **36%** | **90%** | **95%** | **6.90%** | **0%** | **100%** |

**Supplementary Table S2**

**Table S2.** Assembly statistics for the 19 *S*. Infantis genomes. Columns: Isolate ID, number of contigs ≥200 bp, total assembly length (bp), N50 (bp), N75 (bp), N90 (bp), longest contig (bp), shortest contig (bp), average contig length (bp), GC content (%), AuN, and I50.

| **SAMN** | **TotSeqs** | **TotLen** | **N50** | **N75** | **N90** | **I50** | **GC** | **Avg** | **Min** | **Max** | **AuN** |
| --- | --- | --- | --- | --- | --- | --- | --- | --- | --- | --- | --- |
| **SAMN11310164.fa** | 51 | 4888820 | 165881 | 104421 | 59211 | 7 | 52.2 | 95859.22 | 481 | 835311 | 291155 |
| **SAMN11310165.fa** | 75 | 4929300 | 159294 | 65960 | 42057 | 10 | 52.1 | 65724 | 473 | 559840 | 195874 |
| **SAMN24966732.fa** | 80 | 4912010 | 147148 | 74452 | 39087 | 9 | 52.16 | 61400.12 | 473 | 522060 | 211596 |
| **SAMN24966733.fa** | 87 | 4898761 | 106891 | 69378 | 32994 | 12 | 52.07 | 56307.6 | 432 | 685388 | 208602 |
| **SAMN24966734.fa** | 81 | 5060290 | 168430 | 71817 | 43583 | 7 | 52.04 | 62472.72 | 207 | 684405 | 275255 |
| **SAMN24966735.fa** | 60 | 5096234 | 405580 | 112300 | 62508 | 4 | 52.07 | 84937.23 | 234 | 1368835 | 569207 |
| **SAMN24966736.fa** | 60 | 5128038 | 247947 | 136430 | 55979 | 5 | 52.07 | 85467.3 | 358 | 1195119 | 441496 |
| **SAMN24966737.fa** | 62 | 4983346 | 206713 | 123223 | 55091 | 6 | 52.13 | 80376.55 | 315 | 1071794 | 396294 |
| **SAMN24966739.fa** | 48 | 5027764 | 263795 | 152095 | 63350 | 5 | 52.12 | 104745.08 | 473 | 1195119 | 463970 |
| **SAMN24966740.fa** | 54 | 5001891 | 227014 | 129222 | 61501 | 7 | 52.1 | 92627.61 | 473 | 1071806 | 373230 |
| **SAMN24966741.fa** | 47 | 4716272 | 225472 | 98528 | 53002 | 7 | 52.11 | 100346.21 | 336 | 676603 | 291414 |
| **SAMN24966742.fa** | 87 | 5029190 | 158915 | 74663 | 45639 | 9 | 52.11 | 57806.78 | 201 | 476574 | 209632 |
| **SAMN24966743.fa** | 29 | 4806238 | 694304 | 257639 | 152795 | 2 | 52.11 | 165732.34 | 346 | 1779362 | 886771 |
| **SAMN24966744.fa** | 60 | 4965921 | 263795 | 129217 | 55091 | 5 | 52.16 | 82765.35 | 315 | 908128 | 369997 |
| **SAMN24966745.fa** | 57 | 4982361 | 203759 | 119737 | 57386 | 6 | 52.13 | 87409.84 | 358 | 1071796 | 401615 |
| **SAMN24966746.fa** | 72 | 4899568 | 193941 | 80680 | 47100 | 8 | 52.15 | 68049.56 | 358 | 526267 | 227788 |
| **SAMN24966747.fa** | 41 | 4675204 | 216911 | 152104 | 75045 | 6 | 52.28 | 114029.37 | 358 | 1056326 | 409287 |
| **SAMN24966748.fa** | 51 | 5009130 | 269483 | 129222 | 62502 | 5 | 52.12 | 98218.24 | 437 | 1071824 | 433792 |
| **SAMN24966749.fa** | 109 | 4915377 | 123330 | 56539 | 28787 | 11 | 52.11 | 45095.2 | 366 | 535654 | 169952 |

**Supplementary Figure S1**

**Figure S1.** Pairwise core-genome SNP distances among 19 Lebanese *S*. Infantis isolates.

The heatmap shows pairwise core-genome SNP distances between all 19 *S*. Infantis isolates included in this study. Rows and columns correspond to the same isolates (labels on the axes). Colors indicate the magnitude of the distance, according to the scale bar (from blue = low distance to red = high distance, up to ~2000 SNPs). Hierarchical clustering based on these distances is shown as dendrograms above and to the left of the matrix. The central blue/purple block corresponds to the main Lebanese cluster with short distances between isolates (≤167 SNPs), whereas warmer colors at the periphery indicate more distantly related isolates
